# Reorientation of the SUMOylation landscape and altered L3mbtl2-mediated transcriptional activity in cancer-associated muscle contractile dysfunction

**DOI:** 10.1038/s41420-026-03337-y

**Published:** 2026-09-09

**Authors:** Luis Vincens Gand, Mugeng Li, Katharina Brandt, Baoyu Zhou, Amel Nassar-Grioua, Julien Gondin, Edgar Becker, Andreas Pich, Theresia Kraft, Mamta Amrute-Nayak, Arnab Nayak

**Affiliations:** 1 Institute of Molecular and Cell Physiology, Hannover Medical School, Hannover, Germany; 2 Institut NeuroMyoGène (INMG) Physiopathologie et Génétique du Neurone et du Muscle (PGNM) UMR CNRS 5261 – INSERM U1315, Lyon, France; 3 Institut of Toxicology, Hannover Medical School, Hannover, Germany; 4 Present Address: Faculty of Sport and Health Sciences, University of Jyväskylä, Jyväskylä, Finland

**Keywords:** Cell signalling, Chromatin

## Abstract

Cachexia is a debilitating muscle-wasting disorder associated with a high mortality rate in cancer patients. However, the molecular mechanisms of muscle contractile dysfunction underlying cancer-induced cachexia (CIC) remain poorly characterized. Here, we demonstrated that CIC reorients global SUMOylation in skeletal muscle cells and alters the stability of various SUMO machinery components, particularly SUMO isopeptidases. The non-canonical polycomb repressor protein L3mbtl2 was among the predominant proteins with enhanced SUMOylation level in CIC. Surprisingly, in contrast to previous notions, we found that L3mbtl2 activates transcription of a large cohort of genes regulating muscle contraction. Mechanistically, L3mbtl2 associates with Ash2L, a component of the SET1/MLL histone methyltransferase complex. Increased SUMO modification of L3mbtl2 in CIC leads to partitioning of Ash2L from its target genes, resulting in impaired calcium handling, sarcomere disorganization and impeded muscle cell contractile properties. Our findings reveal an unprecedented connection between SUMO and CIC, a paradoxical SUMO-associated transcriptional activator function of L3MBTL2 and hold potential for developing therapeutic interventions to ameliorate CIC.

## Introduction

Accurately organized sarcomeres and efficient calcium handling process are among the critical prerequisites for proper muscle contraction enabling force generation to bear loads and drive movements. Imprecise sarcomere assembly, dysfunction of the contractile proteins, or impaired calcium handling process can result in defective muscle function, commonly observed in muscle wasting disorders. Cachexia is a multifactorial muscle wasting syndrome characterized by severe involuntary skeletal muscle loss- by approximately 30% of their pre-illness level- and occurs in up to ~80% of advanced cancer patients, contributing to mortality in over 30% cases [1–3]. Cancer-secreted pro-inflammatory cytokine-induced nuclear factor kB (NF-kB)-MuRF1 pathway and the metal-ion transporter ZRT- and IRT-like protein 14 (ZIP14) are among the most well-characterized pathways leading to cachexia, or cancer-induced cachexia (CIC) [4, 5]. Small-molecule inhibitors targeting MuRF1 and the epigenetic regulator BRD4 (bromodomain-containing protein 4) in cell culture and mouse model showed attenuation of skeletal muscle atrophy to some extent [6, 7]. Recently, GDF15 (growth differentiation factor 15)-neutralizing antibody was shown to mitigate cancer cachexia by improving muscle loss [8]. Despite these seminal studies, treatment for cachexia remains a challenge due to the lack of any single effective strategy capable of ameliorating the cachectic conditions in patients. This highlights our limited understanding of the molecular mechanisms underlying cachexia.

Posttranslational modification of proteins by covalent addition of SUMO (small ubiquitin-like modifier), known as SUMOylation, is a fundamental molecular mechanism regulating diverse cellular function in metazoan [9–18]. SUMO E1 (SAE1/Uba2), E2 (Ubc9) and E3 enzymes mediate the covalent attachment of SUMO moieties—SUMO1 and highly similar SUMO2/3 isoforms in mammals -to a ε-acceptor lysine of target proteins. SUMO-specific isopeptidases (Senps) remove SUMO groups from substrate proteins and are key determinants of the dynamic SUMOylation process. Although the role of the SUMO pathway in regulating muscle physiology and CIC is poorly defined, it is an emerging area of biology. Metazoan cells utilize the SUMO machinery to rapidly respond to external challenges, particularly during oxidative stress and heat shock [19, 20]. Given that cancer induces oxidative stress [21, 22], we hypothesize a potential yet unexplored, connection between SUMO and CIC.

The expression of *homeobox* (*HOX*) genes - essential for regulating the early stages of metazoan development—is tightly controlled by opposing epigenetic mechanisms mediated by Trithorax (TrxG) and Polycomb (PcG) group of proteins [23–26]. Both TrxG and PcG proteins play crucial transcriptional roles in adult metazoan life. The mixed-lineage leukemia histone methyltransferase complex (SET1/MLL) belongs to the TrxG group of epigenetic modifiers, and consists of a core catalytic subunit (MLL1-4 or the SET1 isoforms SET1A-B), which is further decorated with the WRAD module (WDR5, RbBP5, ASH2L, and DPY30) [27, 28]. This transcription activating epigenetic modifier ensures H3K4me2 and H3K4me3 epigenetic modifications on its target genes. On the contrary, lethal malignant brain tumor like 2 (L3mbtl2) has been identified as a crucial subunit of the non-canonical polycomb repressive complex 1.6 (PRC1.6) [29], which consists of several subunits such as PCGF6. PRC1.6 typically binds to histone tails, acts in concert with PRC2 (polycomb repressive complex 2) and establishes a repressive chromatin state [30]. However, potential involvement of SET1/MLL and polycomb repressor proteins complexes in CIC remain largely unsettled.

In this study, we investigated the link between SUMO modifications and L3mbtl2 function in CIC context. We observed reorientation of global SUMOylation in CIC. We identified that increased SUMOylation of L3mbtl2 in CIC led to chromatin partitioning of Ash2l away from muscle-specific gene, impairing calcium handling process, and causing sarcomere disorganization. Altogether, these changes ultimately resulted in defective muscle contraction.

## Results

### Altered global protein SUMOylation in cancer-induced cachexia

First, we assessed potential changes in global SUMOylation in CIC. CIC was induced by incubating differentiated muscle cells (myotubes) with cancer-secreted pro-inflammatory cytokines TNFα and IFNγ [31, 32] (Fig. S1A). As hallmark of cachexia induction, we evaluated myosin heavy chain (MyHC-IId) protein levels. TI (TNFα and IFNγ) treatment led to reduced MyHC-IId protein levels consistent with cachexia (Figs S1B and S7A). After confirming cachexia induction, we examined SUMOylation levels of proteins in cachectic myotubes. Compared to control lysates, we observed an increase in higher molecular weight species of SUMO2/3-conjugated proteins- but not SUMO1-modified proteins- in cachectic lysates (Figs. S1B and S7B, C). A similar observation was confirmed in human skeletal myoblast-derived primary muscle cells (Figs. S1C and S7D–F). To validate it, we harvested extensor digitorum longus muscles (EDL) the preclinical cancer cachexia model of C26 (colon 26 adenocarcinoma)-bearing mice [33, 34] and confirmed a reduction in MyHC-IId protein expression, indicative of cachexia development. Interestingly, consistent with our muscle cell model, global SUMO2/3-conjugation was elevated in muscle tissues from cachectic mice compared to wild-type littermates. (Figs. S1D and S8), suggesting that reorientation of global SUMOylation is associated with CIC.

**Fig. 1:**
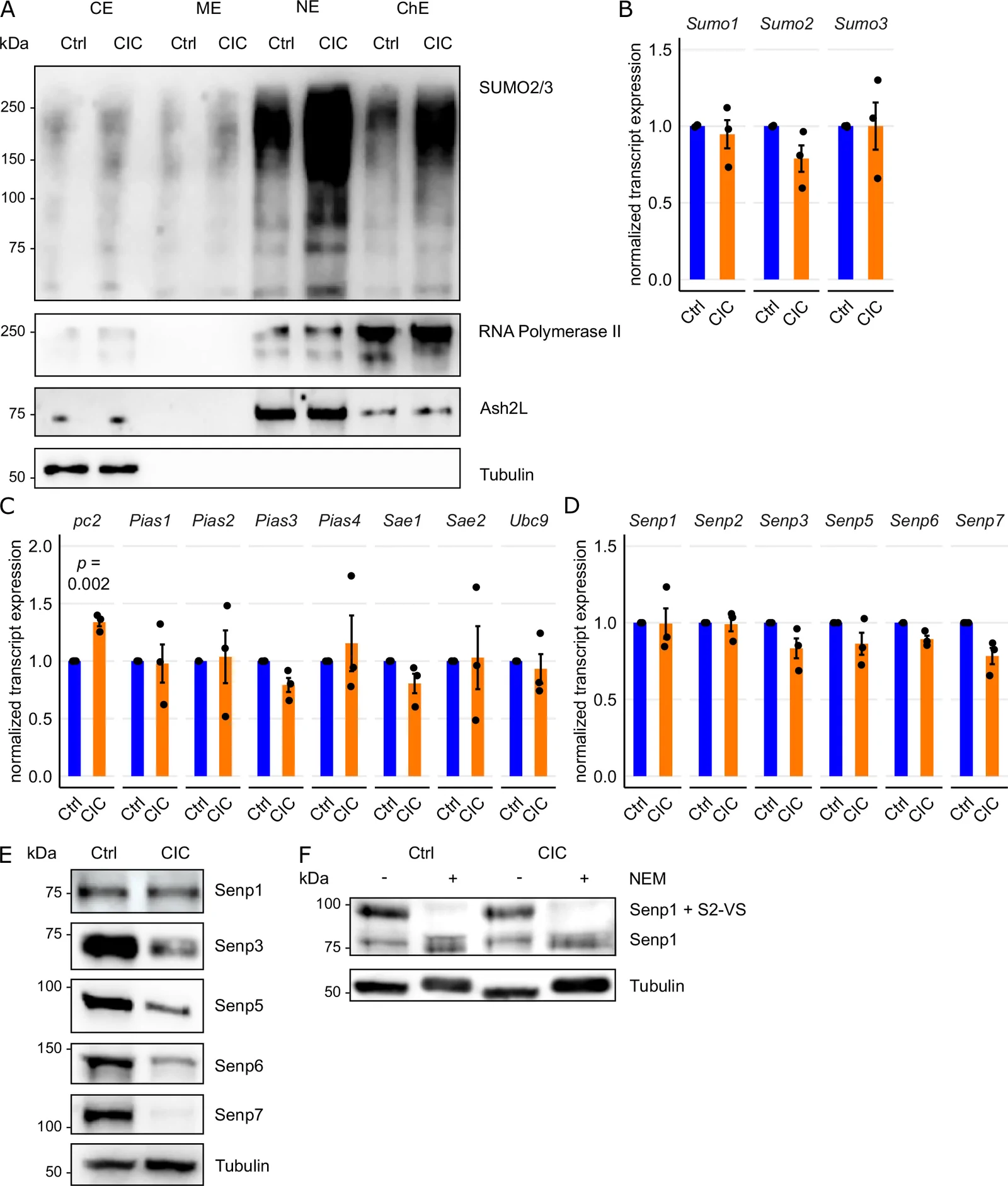
CIC alters SUMO-conjugated protein landscpe in muscle cells. **A** Immunoblot from subcellular fractions from control and cachectic differentiated muscle cells (myotubes) probed against indicated antibodies. CE cytosolic extract, ME membrane-bound extract, NE nuclear soluble extract, ChE Chromatin-bound extract. Ash2L, mainly present in soluble NE shows brighter signal from the NE, RNA Polymerase II (Pol II) most predominantly present in chromatin bound fraction shows higher Pol II signal in chromatin. The cytosolic protein marker tubulin was mostly observed in CE. **B**–**D** RT-qPCR from control and cachectic myotubes showing transcript expression of components of SUMO conjugation machinery, displayed as mean ± SD, *N* = 3 biological replicates with technical replicates in each experiments, statistical significance tested with unpaired two-sided t-test. **E** Same as in (**A**), but whole cell extracts were probed against indicated antibodies, with anti-tubulin antibody as loading control. **F** Immunoblot with control and cachectic samples incubated with SUMO2-VS with or without NEM probed against indicated antibodies. NEM inactivates catalytic cysteine of Senp, thus no Senp-SUMO adduct will form. NEM N-ethylmaleimide, VS vinyl sulfone.

In subcellular fractionation experiments, SUMO2/3-conjugated proteins were enriched in both soluble nuclear (NE) and chromatin-bound (ChE) fractions and their levels increased further under CIC condition (Figs. 1A and S6). Upon CIC induction, we could detect increased SUMO-conjugates in cytosolic (CE) and of membrane-bound (ME) fractions as well, however extent of overall SUMOylation in these two fractions were low compared to NE and ChE. To investigate the potential cause of increased SUMOylation in CIC, we examined whether transcription of genes encoding components of the SUMO machinery was altered. RT-qPCR (reverse transcription quantitative PCR) assays showed no significant changes in the expression of all major mammalian SUMO isoform transcripts in CIC (Fig. 1B). Furthermore, genes encoding SUMO conjugation machinery SUMO E1, E2, E3 enzymes and Senps were all unchanged on transcript level, except for *pc2* (Polycomb 2) which showed a modest increase of 37% (*p* = 0.002) (Fig. 1C). Interestingly, SUMO isopeptidases Senp3, Senp5, Senp6 and Senp7 were reduced at protein level, whereas Senp1 levels remained unaltered at both the transcriptional and protein levels (Figs. 1D, E and S9A) in CIC. Since Senp1 protein expression was not reduced in CIC, we further assessed its catalytic activity. Senp1 formed a stable adduct with SUMO2-VS (vinyl sulphonate) in both control and cachectic cells, indicating that Senp1 catalytic activity was unaltered in CIC (Figs. 1F and S9B: additional details in M &M). Taken together, CIC increased SUMO2/3-modification of proteins, particularly those localized in the nucleus. It is likely that the overall decrease in Senp protein levels might have contributed to increased protein SUMOylation in CIC.

### Mass spectrometric screen of SUMO2/3-conjugated proteins in CIC

**Fig. 2:**
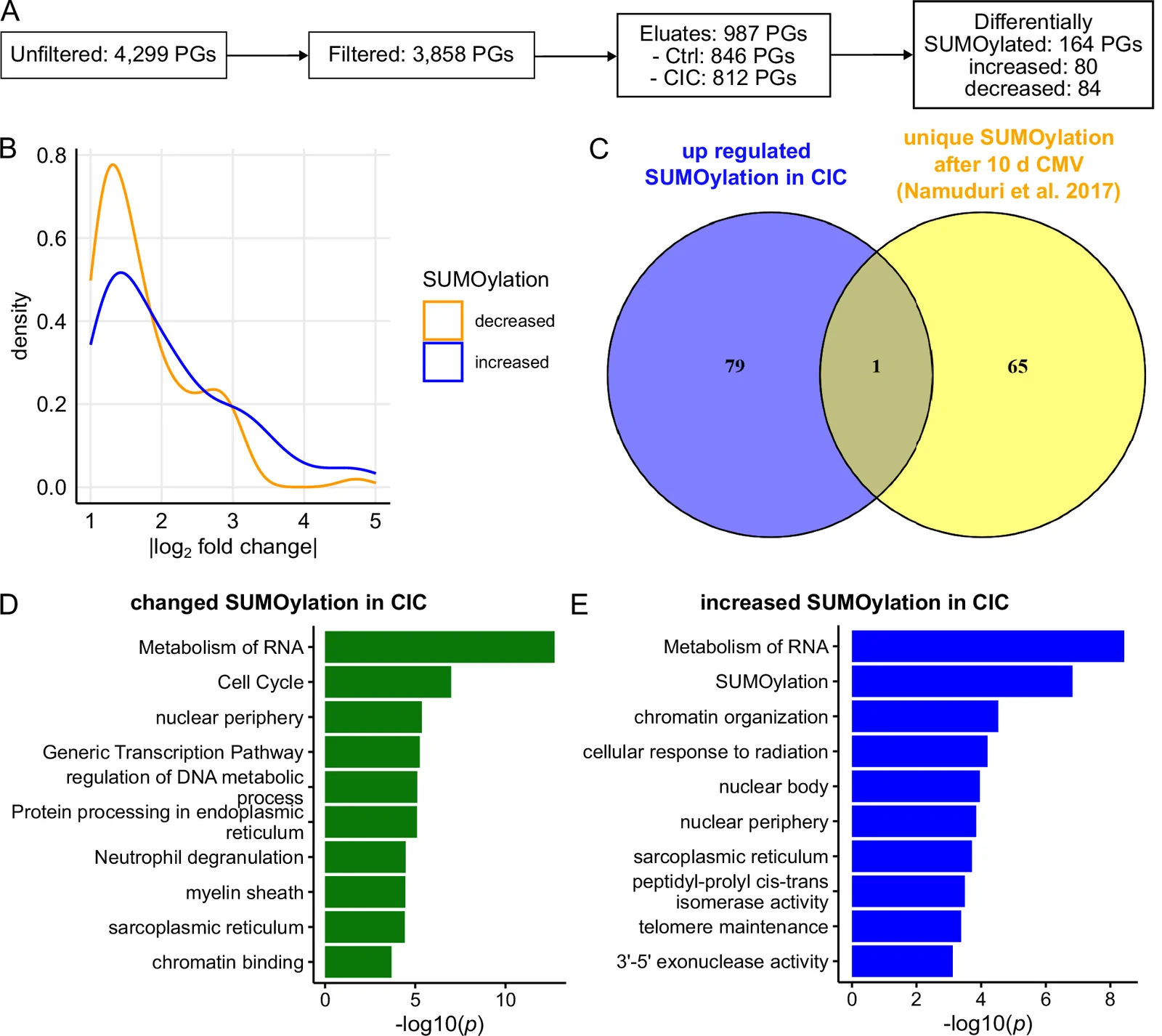
Mass spectrometric (MS) proteomic analysis. **A** Bioinformatics pipeline of SUMO2/3 modified proteins identified by MS, *N* = 3 biological replicates. Filtering procedure detail is mentioned in method section. **B** Density plot showing the modulus of change in SUMO2/3-modification in cachectic myotubes. Log_2_ fold changes represents negative values for PGs with decreases SUMOylation (orange). Log_2_ fold changes represents positive values for PGs with increased SUMOylation (blue). **C** Venn diagram showed comparison of our study with Namuduri et al. [36]. **D**, **E** MetaScape analysis of all proteins with regulated SUMO2/3 modification in CIC. **E** Proteins with increased SUMO2/3 modification were analyzed.

To identify proteins with altered SUMOylation levels in CIC, we performed large-scale endogenous SUMO2 immunoprecipitation [35] followed by quantitative mass spectrometric (MS) analysis. We observed 80 protein groups (PGs) with increased SUMOylation levels in CIC, while 84 PGs showed a decrease in SUMO-conjugation (Fig. 2A and Supplementary Table 1). Analysis of the magnitude of change in SUMO2/3-conjugated proteins revealed a greater overall increase in PGs with elevated SUMOylation than PGs with decreased SUMOylation, confirming our initial observation of an overall trend towards increased SUMOylation in CIC (Fig. 2B).

We compared the subset of proteins with increased SUMOylation in CIC to a previously published analysis of protein SUMOylation following controlled mechanical ventilation-induced stress in rat skeletal (diaphragm) muscle and found only one overlap between the conditions [36] (Fig. 2C), suggesting that distinct PGs are affected in CIC. To better understand the physiological role of SUMOylation changes in CIC, we performed ontology analysis of all 164 PGs with altered SUMOylation using MetaScape [37]. Several highly enriched pathways were related to the nuclear processes, i.e. “metabolism of RNA”, “cell cycle”, “positive regulation of DNA replication” and “chromatin binding” (Fig. 2D). Interestingly, the “sarcoplasmic reticulum”, a muscle-specific compartment of the endoplasmic reticulum critical for calcium transient and muscle contraction, was also among the highest ranking ontologies (Fig. 2D). Further analysis of the 80 PGs with increased SUMOylation identified highly enriched terms unique to this subset of PGs, such as “SUMOylation”, “chromatin organization” and stress-related pathways like “cellular response to radiation” (Fig. 2E).

### L3mbtl2 is a novel regulator of skeletal muscle function

L3mbtl2 was among those proteins with predominantly increased SUMO2/3-conjugation in CIC. We validated this by immunoprecipitating endogenous SUMO2/3 followed by immunoblotting against L3mbtl2. This showed increased SUMOylation of L3mbtl2 in cachectic lysates compared to control (Fig. S2A, B).

**Fig. 3:**
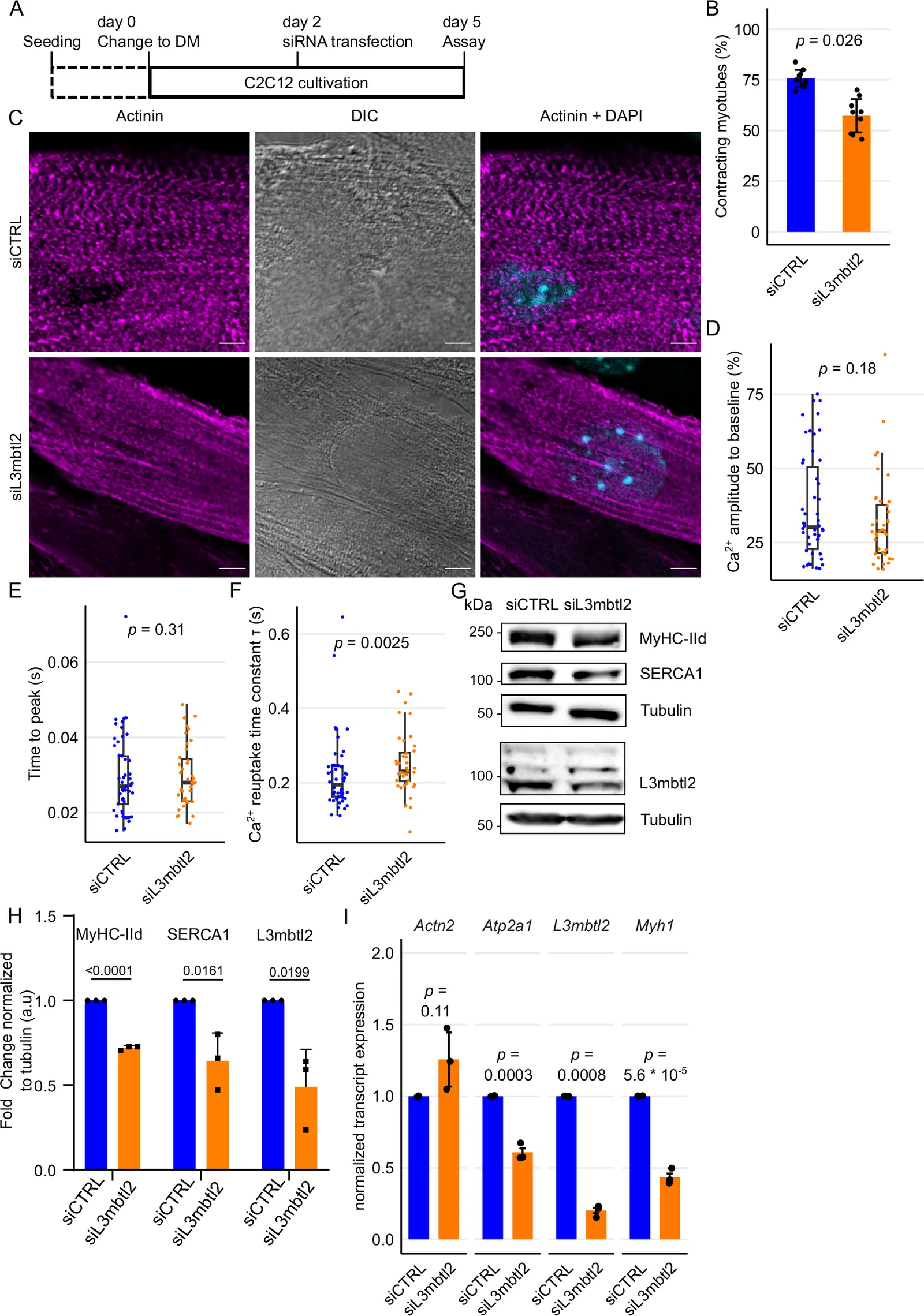
L3mbtl2 modulates function physiology. **A** Scheme illustrating siRNA transfection procedure. **B** Percentage of contracting myotubes when placed under electrical stimulation at 40 V, 1 Hz and 4 ms pulse duration. 728 control and 745 *L3mbtl2*-depleted myotubes from 3 biological replicates were analyzed in total, statistical significance was tested by an unpaired two-sided t-test. **C** Confocal images showing immunofluorescence staining against alpha-actinin protein (marker for sarcomeric Z-discs) in control and *L3mbtl2*-depleted myotubes, scale bar is 5 µm. **D** Ca^2+^ amplitude (peak) compared to baseline in percentage, **E** time between electric stimulus until Ca^2+^ peak (maximal Ca^2+^ release) is reached, and **F** single exponential decay of Ca^2+^ relaxation time constant (τ) in seconds (Ca^2+^ re-uptake time). **D**–**F** In total, we analyzed 115 control and 107 *L3mbtl2*-depleted cells from 4 biological replicates. Significance was tested using Mann-Whitney-U-test. **G** Immunoblots from control and L3mbtl2-depleted myotubes probed against indicated antibodies *N* = 3 biological replicates. **H** ImageJ analysis shows relative amount of indicated protein signals (nornalized against tubulin) from Ctrl and CIC conditions from three biological experiments. siCTL value was set as 1. Data represent the mean (±SD) of three biological experiments. **I** RT-qPCR analysis of indicated transcripts in control and L3mbtl2-depleted myotubes, *N* = 3 biological replicates, statistical significance tested by an unpaired two-sided t-test.

In an entirely different context, increased SUMOylation of L3mbtl2 on lysine 675 and 700 has been shown associated with its transcriptional repressive function [38]. Moreover, defective gene expression of *Atp2a1* (encoding sarcoplasmic/endoplasmic reticulum Ca^2+^-ATPase pump, Serca1) and *Myh1* (encoding the molecular motor protein myosin heavy chain, MyHC-IId) has been reported in CIC as among the initial causes for defective calcium transient pathway sarcomere disorganization, ultimately resulting in impaired muscle cell’s contraction [32]. Thus, we hypothesized that increased SUMOylation of L3mbtl2 might suppress the transcription and expression of several muscle-specific genes in CIC such as *Atp2a1* and *Myh1*. To test this idea, we first assessed (1) whether L3mbtl2 has functional relevance in muscle cells and (2) whether L3mbtl2 represses muscle specific genes. First, we investigated the contractile ability of myotubes depleted of *L3mbtl2* following electric stimulation (Fig. 3A, B). An electric stimulus mimicking a neural action potential depolarizes muscle cell membrane, triggering the excitation-contraction coupling (ECC) process that ultimately induces Ca^2+^-mediated acto-myosin cross bridge cycling within the sarcomeres, resulting in muscle contraction. In control treatment (siCTRL-transfected muscle cells) 75% of cells exhibited periodic contraction. However, *L3mbtl2* depletion led to a significant 27% drop in the proportion of contracting cells (55% contracting cell, *p* = 0.026, Fig. 3B, Video S1 and Figs. S3). In addition, contractile cohorts of L3mbtl2 knockdown cells exhibited less discrete and less regular movement (contraction) over time compared to control cells (Fig. S3, middle panel). These results further support that L3mbtl2 deficiency led to apparent loss of contraction in 27% of cells and defective contraction in remaining cells that retained some degree of contractile activity upon L3mbtl2 knockdown. To investigate the underlying cause of defective contractile ability, we stained paraformaldehyde-fixed control and *L3mbtl2*-depleted myotubes for the sarcomeric Z-disc protein alpha-actinin. Control transfected cells presented a striated pattern indicative of well-organized sarcomeres. In contrast, *L3mbtl2*-depleted cells displayed partial loss of prominent sarcomeric striations (Fig. 3C), suggesting that L3mbtl2 is important for proper sarcomere organization. Given that calcium (Ca^2+^) handling is another critical feature of muscle contraction, we examined whether L3mbtl2 also affects this process. Upon electric stimulation, control cells exhibited a detectable Ca^2+^ transient characterized by a strong Ca^2+^ release from the sarcoplasmic reticulum (SR) via ryanodine receptor (Ryr1) followed by Ca^2+^ reuptake (fluorescence decay) by Serca1 back into the SR [39]. Compared to control cells, *L3mbtl2*-depleted cells showed no significant difference in Ca^2+^ release properties, Ca^2+^ amplitude (30.1 ± 4% in siCTRL vs. 28.9 ± 1.5% in siL3mbtl2, *p* = 0.18) and time to peak (27 ± 1.3 ms in siCTRL vs. 28 ± 2.4 ms in siL3mbtl2, *p* = 0.31) were unchanged (Fig. 3D, E). However, *L3mbtl2*-depleted cells exhibited a significant increase in the Ca^2+^ reuptake time constant (195 ± 22 ms in siCTRL vs. 230 ± 28 ms in siL3mbtl2, *p* = 0.0025) (Fig. 3F), indicating defect in calcium handling. As several contraction-related parameters were impaired in *L3mbtl2*-depleted myotubes, we investigated the protein levels of the molecular motor proteins responsible for skeletal muscle contraction, MyHC-IId, as well as Serca1, which is responsible for Ca^2+^ reuptake. Strikingly, both MyHC-IId and Serca1 were strongly reduced at protein levels in *L3mbtl2*-depleted cells (Fig. 3G, H), most likely due to reduced transcriptional activity of *Myh1* and *Atp2a1* genes (Fig. 3I). Taken together, these findings suggest that, contrary to its established role as a gene repressor, L3mbtl2 acts as a transcriptional activator of key skeletal muscle-specific genes. Furthermore, depletion of *L3mbtl2* phenocopies CIC in skeletal muscle cells, as evidenced by reduced expression of *Myh1* and *Atp2a1* at both transcript and protein levels, impairing sarcomere organization and compromising Ca^2+^ transient, ultimately leading to defective contraction.

### L3mbtl2 has a dual transcriptional role in skeletal muscle

To investigate the potential role of L3mbtl2 in regulating global muscle-specific transcription, we performed RNA sequencing and compared control and *L3mbtl2*-depleted cells. Principle component analysis (PCA) analysis demonstrated that control and *L3mbtl2*-depleted samples formed distinct well-separated clusters (Fig. S4A). *L3mbtl2* depletion resulted in 1,010 differentially expressed genes in myotubes (Fig. S4B). Of these, 665 genes were upregulated, while 345 genes were downregulated (Fig. S4B, C). These findings indicate that L3mbtl2 is contributing to activate transcription of 345 genes, which is notable because L3mbtl2 is typically characterized as a transcriptional repressor [40]. Consistent with this, RNA-seq analysis showed reduced expression of *Atp2a1* and *Myh1* in *L3mbtl2*-depleted myotubes (Fig. S4C). MetaScape analysis revealed that the downregulated genes were highly enriched in skeletal muscle-related terms, such as ‘myofibril’, ‘sarcoplasm’, and ‘A band’ (Fig. S4D). Genes up regulated by *L3mbtl2* knockdown were enriched for terms such as ‘muscle contraction’, ‘muscle structure development’, and ‘muscle system process (Fig. S4E). Muscle-specific terms were highly enriched in both upregulated and down regulated gene sets. Therefore, we further analyzed differentially expressed genes related to these ontologies. Chord plot analysis demonstrated that genes generally expressed in striated muscle were predominantly downregulated following *L3mbtl2* depletion. Genes specific to fast skeletal muscle were also downregulated, except for specialized myosin heavy chain genes *Myh3*, *Myh8*, and *Myh13*. While cardiac-specific genes were almost exclusively upregulated by *L3mbtl2* depletion (Fig. S5A). We validated L3mbtl2 RNA-seq experiments by performing independent experiments. The expression of skeletal muscle-specific *Casq1, Mylf* and *Tnni2* was downregulated by ~3, ~3.2, ~4.1, respectively (Fig. S5B). Whereas, the expression of cardiac-specific genes was increased by ~5 fold (cardiac *Myl2)* and ~3 fold (*Tnni3*) and in L3mbtl2 depleted cells. These observations confirm and validate the outcomes presented in Figure S5A. Notably, comparison of our previously reported RNAseq dataset from cachectic myotubes [32] with the *L3mbtl2*-depleted RNAseq dataset, revealed a substantial overlap in downregulated genes. Transcription of 90 genes was impaired in both conditions i.e., in CIC as well as with *L3mbtl2* depletion (Fig. S5C). We have also observed increased expression of *MuRF1, Atrogin-1* (Fig. S5D) and decrease in myotubes´ length and diameter (Fig. S5E) in CIC conditions. It further supports induction of cachectic pathway in our study. Chord plot analysis of these common genes showed significant enrichment for terms such as ‘Muscle system process’, Muscle contraction’, and ‘Sarcomere organization’ (Fig. S5F). Several key genes encoding proteins essential for muscle contraction were among the shared down regulated, including *Myh1*, *Atp2a1*, *Mybpc1*, *Casq1*, and *Tpm1*. These results underscore the critical role of L3mbtl2 in proper muscle function.

### L3mbtl2 directly activates *Atp2a1* gene expression via interaction with SET1/MLL complex components

**Fig. 4:**
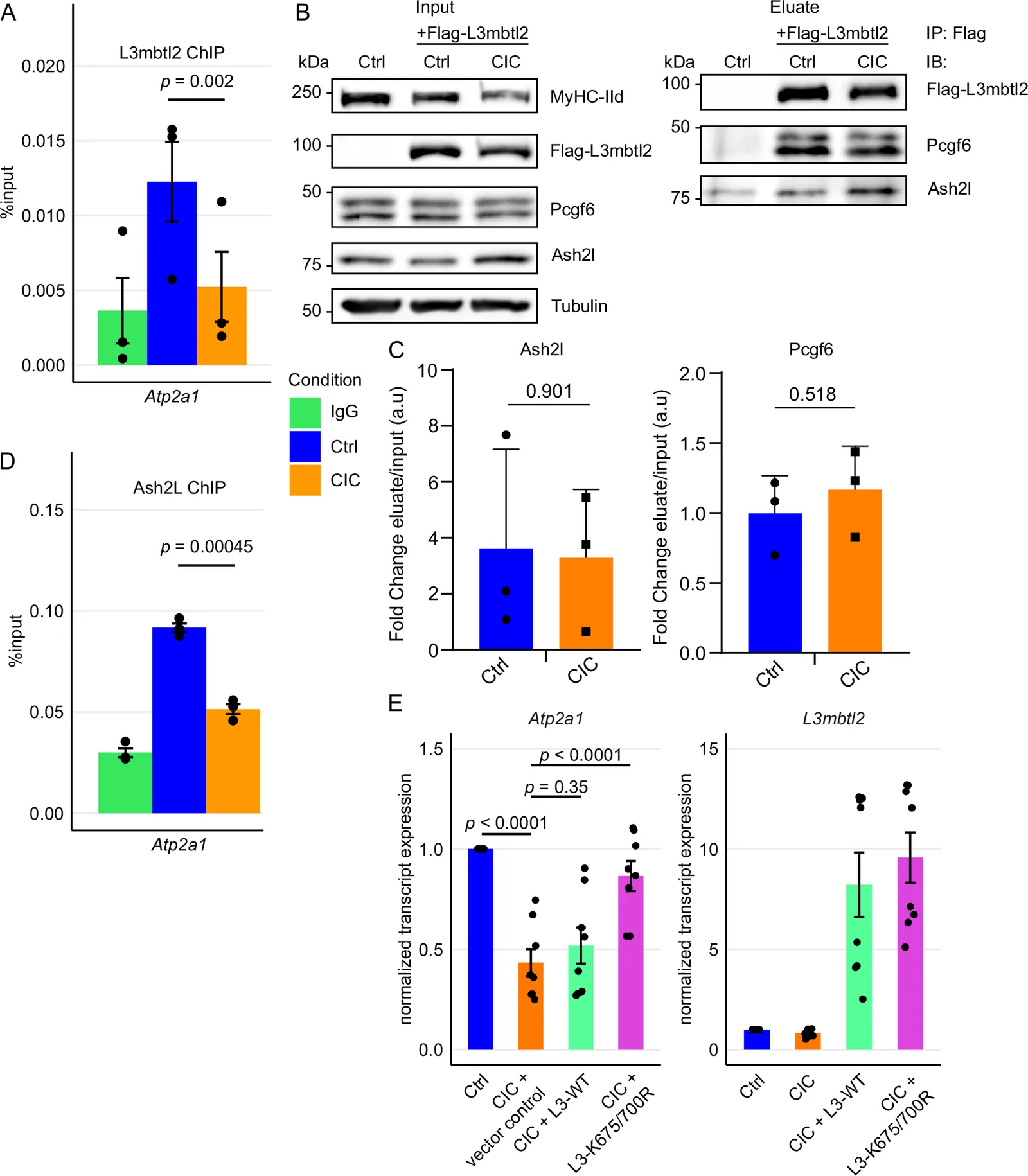
L3mbtl2 is associated with SET1/MLL complex. **A** ChIP assay with antibody specific to L3mbtl2 was performed in Ctrl and CIC myotubes on promoter regions of *Atp2a1* gene, displayed as mean ± SEM from 3 biological replicates and 12 technical replicates, significance tested with two-sided t-test. As a negative ChIP control, we used IgG isotype control antibody. **B** Immunoblot of input lysates (left part) and eluate samples (right part) from Ctrl and CIC myotubes with exogenous expression of Flag-L3mbtl2 after immunoprecipitation probed with indicated antibodies. **C** In this ImageJ analysis, Ash2l elute Ctrl signal was normaized by Ash2l input Ctrl. Similarly, Ash2l elute CIC signal was normaized by Ash2l input CIC. Data represents mean (±SD) of three biological experiments, a.u. arbitrary unit. **D** Same as in (**A**), but anti-Ash2L antibody was used for ChIP. **E** RT-qPCR analysis of Ctrl, and CIC conditions with transiently expressing WT or KR-version of L3mbtl2, *N* = 8 biological replicates with technical replicates in each experiments, statistical significance was tested by an unpaired two-sided t-test.

As L3mbtl2 is a chromatin-associated protein and we observed its crucial regulatory role in skeletal muscle, we next sought to gain detailed insights into the chromatin-related mechanisms underlying its function. To this end, we performed chromatin immunoprecipitation (ChIP) assays in both control and cachectic myotubes and precipitated chromatin complexes by using a specific antibody against L3mbtl2 to precipitate chromatin complexes and selected *Atp2a1* as a model gene. qPCR analysis was performed with primers targeting *Atp2a1* promoter. The specificity of the antibody was validated by a 3.4-fold enrichment of L3mbtl2 protein on the *Atp2a1* promoter in control conditions compared to the IgG isotype antibody (Fig. 4A). Notably, L3mbtl2 occupancy at the *Atp2a1* promoter was significantly reduced by 57% in cachectic conditions relative to control conditions, indicating that L3mbtl2 directly targets *Atp2a1* gene and higher SUMOylation of L3mbtl2 might be associated with its lower chromatin occupancy.

Furthermore, we included wild-type Flag-L3mbtl2 (L3-WT) or a mutant form in which both SUMO acceptor lysines (K675 and K700) [38] are mutated to arginines (i.e., R675 and R700 or K675/700R), i.e., rendering L3mbtl2 incapable of SUMOylation. ChIP assays show a ~2,4 fold more chromatin enrichment of the SUMO site mutant (Flag-L3mbtl2-K675/700 R), compared to the wild type Flag-L3mbtl2 in control condition (Fig. S10A). In cachectic cells, chromatin enrichment of the transiently expressed wild type Flag-L3mbtl2 was reduced by ~2 fold (on *Atp2a1* promoter). Interestingly, chromatin enrichment remained nearly unchanged in case of Flag-L3mbtl2-K675/700R in CIC (Fig. S10A–C), highlighting the relevance of L3mbtl2 SUMO sites in CIC.

To further elucidate the relevance of increased L3mbtl2 SUMOylation in this context, we transfected cells with Flag-L3mbtl2 and performed immunoprecipitation assays using an anti-Flag antibody on control and cachectic cell lysates. Flag-L3mbtl2 was specifically immunoprecipitated from transfected, but not untransfected samples, confirming assay specificity (Fig. 4B). The core subunit of PRC1.6, Pcgf6 co-immunoprecipitated with L3mbtl2, in both control and cachectic myotubes, with no observable difference between conditions (Fig. 4B, right panel, Fig. 4C). Our previous work demonstrated that H3K4me3 levels are reduced at the *Atp2a1* promoter in CIC [32]. Additionally, the SET1/MLL complex subunit Ash2l function was identified as a critical regulator of muscle-specific gene [41]. We therefore examined association between L3mbtl2 with Ash2l. Interestingly, L3mbtl2 was associated with Ash2l in control muscle cells, which did not change significantly in cachectic lysates (Fig. 4B, right panel, Fig. 4C). ChIP assays using Ash2l antibody revealed a 3.1-fold enrichment of Ash2l at the *Atp2a1* promoter compared to IgG isotype control antibody, which was significantly reduced by 44% in CIC (Fig. 4D). Collectively, these findings suggest that enhanced SUMOylation of L3mbtl2 in CIC may result in detachment of both L3mbtl2 and Ash2l from its target gene, thereby reducing *Atp2a1* expression.

To further elucidate the role of SUMOylation in regulating L3mbtl2, we transfected muscle cells with either wild-type L3mbtl2 (L3-WT) or L3mbtl2-K675/K700R) [38]. Following transfection, CIC was induced. *Atp2a1* transcript levels were downregulated by ~2.2-fold in cachectic condition. Interestingly, L3mbtl2- K675/700R variant, but not the wild type, could partially (by 82%) yet significantly rescue *Atp2a1* transcript levels in cachectic myotubes (Fig. 4E). Altogether, these findings provide direct evidence linking SUMOylation of L3mbtl2 to its role in CIC.

## Discussion

Here, we demonstrated an unprecedented role for SUMOs in muscle contractile dysfunction associated with CIC. An overall trend of upregulated global SUMOylation was observed in CIC. However, our endogenous SUMO proteomics reveals a more nuanced outcome. The numbers of proteins with higher *vs*. lower SUMOylation level were comparable. Nevertheless, the number of proteins with increased SUMOylation was greater than those with decreased SUMOylation, explaining the overall direction of increased SUMOylation in CIC (Fig. 2B). A possible reason of increased SUMOylation was mainly due to lower protein expression of Senp3, 5, 6 and 7 in CIC. CIC did not affect the catalytic activity of Senp1, whose expression did not follow the other Senps. Previous reports showed increased SUMOylation of proteins in rat diaphragm muscle upon mechanical ventilation [36]. When compared to our report, there was only one SUMOylated protein overlap (Rpl14) between the two studies (Fig. 2C), reinforcing the idea that distinct sets of protein SUMOylation are most likely stress-specific.

An attractive feature of this study is that a candidate protein with increased SUMOylation, i.e., L3mbtl2 activated a large number (345) of muscle-specific genes, thereby establishing a paradoxical functional importance of L3mbtl2 in muscle cells. In a completely different cellular context (HEK293 cells), a previous study demonstrated that SUMOylation of L3mbtl2 facilitates repression of PRC1.6-target genes [38]. Compared to our study, the type of target genes examined in that report was also different. In contrast to its known transcriptional repressive function, L3mbtl2 was identified as a transcriptional activator in our current study. *Atp2a1* and *Myh1* were among the most prominent genes activated by L3mbtl2. Besides, L3mbtl2 phenocopied CIC, as we observed a large overlap between our CIC RNAseq and L3mbtl2 RNAseq datasets (90 commonly down regulated genes). Our in-depth mechanistic assessment revealed that L3mbtl2´s transcriptional activating function was partly due to its association with SET1/MLL histone methyltransferase complex component, Ash2l. Increased SUMOylation of L3mbtl2 in CIC hampered the transcriptional function of L3mbtl2. We propose that under normal condition L3mbtl2 interacts with Ash2l in muscle cells. Association of L3mbtl2 and Ash2l remained unchanged (Fig. 4B, C) in CIC. However, increased L3mbtl2 SUMOylation reduced chromatin association of both L3mbtl2 and Ash2l from its target gene promoter *Atp2a1* (Fig. 4A, D). Our candidate-based ChIP assays support this notion, as we found reduced chromatin association of both L3mbtl2 and Ash2L in CIC. This is a possible explanation for the observed defective *Atp2a1* transcription that led to calcium handling defect and ultimately impaired muscle cells’ contraction observed in cachectic myotubes. Higher SUMOylation of L3mbtl2 does not affect its association with other known interacting partner such as Pcgf6. SUMO-conjugation site mutant of L3mbtl2 additionally showed higher chromatin occupancy on the *Atp2a1* promoter in both control and cachectic conditions (Fig. S10). Most interestingly, we observed a significant rescue of *Atp2a1* expression in cachectic muscle cells in the presence of the SUMO-conjugation site mutant of L3mbtl2, indicating a direct link between L3mbtl2 SUMOylation and its impact on muscle-specific gene expression. Considering previous reports and our observation in this study, we propose that L3mbtl2 may exists in distinct complexes in muscle cell: one fraction is associated with PRC1.6 complexes (transcriptional repressor) and another fraction with SET1/MLL complex component Ash2l (transcriptional activator). Site-specific and cellular context-dependent regulation of L3mbtl2 likely determines its final transcriptional function. Supporting this notion, specific transcriptional regulators such as SENP3 have been reported to be associated with and regulate functionally opposite chromatin regulatory complexes, (e.g. SET1/MLL and PRC1) [42, 43]. The outcome of their action depends on the nature of specific signaling cascades and the cellular microenvironment.

Notably, more than 600 genes were upregulated following *L3mbtl2* depletion, also supporting the role of L3mbtl2’s as a transcriptional repressor in muscle cells. Interestingly, many of the muscle-specific genes that were upregulated are typically expressed in cardiomyocytes where they contribute to cardiomyocyte fate and function. Under L3mbtl2-depleted conditions these genes re-expressed in skeletal muscle cells. This observation suggests that L3mbtl2 is necessary for establishing and maintaining skeletal muscle identity by promoting the expression of skeletal muscle-specific genes, while repressing cardiac muscle-specific genes. Further detailed future investigation is needed to clarify the precise mechanisms involved.

Muscle weakness could happen without muscle atrophy and muscle weakness precedes muscle atrophy during cancer-induced cachexia [44]. Besides, muscle weakness frequently occurs in atrophic muscles with contractile dysfunction [45–49]. Previously, we and other labs have shown that- in addition to reduction in myosin heavy chain protein [50–52], reduced SERCA1 and RyR1 (Ryanodine receptor) proteins in cancer-cytokine-induced and chemotherapy-induced cachectic striated muscle cells are often associated with impaired calcium transient process and contractile defects [32, 53]. Collectively, the aforementioned evidences imply that impaired calcium transient process, deregulation of myosin heavy chain protein level, sarcomere disorganization and contractile defects are among the important physiological signatures associated with CIC. It also suggests that both weakness and atrophy are among the key indicators of muscle dysfunction associated with CIC. In conclusion, we observed an atypical connection of SUMOylation with muscle contractile dysfunction associated with CIC (Figure S11). Restoring balanced SUMOylation level of L3mbtl2 could significantly rescue CIC condition. Thus, future efforts to interfere with SUMOylation of selected candidates, such as L3mbtl2, might be exploited to improve cachexia- a condition for which there are currently no effective therapies.

### Limitations of the study

Although we validated our CIC cell culture model with muscle tissues from cachectic mice, we could not have access to human muscle tissues to validate it further. In addition, we compared our proteomic dataset of mouse species with another proteomic data of rat species. Although they are both employed as rodent models with a high degree of conservation in many basic biological pathways, they are nevertheless distinct mammalian species. We performed the comparison because the published study is somewhat closer to what we have reported in the manuscript i.e, altered muscle SUMOylome under stress condition. Ours is CIC associated stress and the published one was mechanical stress. Extremely low overlap (only one protein – Rpl14) between these two studies suggests that the change in muscle SUMOylation is most likely specific to the nature of stress and underlying pathological changes.

## Material and methods

### Antibodies

For western blotting the following antibodies were used: α-MyHC-IId (1:2,000; Abcam, UK, #ab91506), α-MyHC-IIa (1:1,000, SCBT, #SC-53095), α-SENP1 (1:500, SCBT, #SC-271360), α-SENP2 (1:1,000, abcam, #ab58418), α-SENP5 (1:1,000, Proteintech, #19529-1-AP), α-SENP6 (1:500, SCBT, #SC-100585), α-SERCA1 (1:1,000; Invitrogen, USA, #MA3-912), α-SUMO1 (1:1,000, Cell signaling, #4930), α-SUMO2/3 (1:1,000, MBL, #M114-3), α-Ash2L (1:1,000, Bethyl, #A300-489A), α-RNA Polymerase II (1:1,000, Abcam, #ab5095), α-L3mbtl2 (1:500, Active Motif, #39569), α-Flag (1:1,000, Thermo, #MA1-91878), α-Pcgf6 (1:1,000, Abcam, #A200038), α-MLL (1:1,000, Active Motif, #61295), α-Tubulin (1:15,000; BioCat, Germany, #G098), goat-α-mouse-HRP (1:3,000; Bio-Rad, USA, #172-1011), goat-α-rabbit-HRP (1:3,000; Bio-Rad, USA, #170-6515), rabbit monoclonal α-SENP3 (Cell Signaling Ca#5591), rabbit polyclonal anti-SENP7 (GeneTex Cat#GTX55205). For immunofluorescence the following antibodies were used: α-Actinin-2 (1:100; Abcam, UK, # ab137346), goat-α-rabbit-Alexa488 (Invitrogen, USA, #A-11008). For chromatin immunoprecipitation the following antibodies were used: α-L3mbtl2 (10 µl, Active Motif, #61295), α-Ash2L (10 µg, Bethyl, #A300-489A), rIgG isotype control (10 µg, Cell signaling, #2729). For immunoprecipitation the following antibodies were used: α-SUMO2/3 (8 µg, MBL, #M114-3), mIgG isotype control (8 µg, Invitrogen, #10400 C), α-Flag (10 µg, Thermo, #MA1-91878), rIgG isotype control (10 µg, Cell signaling, #2729).

### Oligonucleotides

The following oligos were used for RT-qPCR:

Actn2 forward: 5’-TGCTGCTATGGTGTCAGAGG-3’

Actn2 reverse: 5’-TGGGGTCATCCTTGTTAAGC-3’

Atp2a1 forward: 5’-AGCTTGACGAGTTTGGGGAG-3’

Atp2a1 reverse: 5’-GCGTTCTTCTTTGCCATCCG-3’

Gapdh forward: 5’-AACTTTGGCATTGTGGAAGG-3’

Gapdh reverse: 5’-ACACATTGGGGGTAGGAACA-3’

L3mbtl2 forward: 5’-GGGAAGACGTCATGAAGGGG-3’

L3mbtl2 reverse: 5’-ACAGTTCCCAAGTTGCACCA-3’

Myh1 forward: 5’-CAAGTCATCGGTGTTTGTGG-3’

Myh1 reverse: 5’-TGTCGTACTTGGGAGGGTTC-3’

Myh2 forward: 5’-GGCCAAAATCAAAGAGGTGA-3’

Myh2 reverse: 5’-CGTGCTTCTCCTTCTCAACC-3’

pc2 forward: 5’-AGCAGCTGATGGGATATCGC-3’

pc2 reverse: 5’-TTGAGCTCATACTGGTGCCC-3’

Pias1 forward: 5’-CCCGGACATAAAGCTGCAGA-3’

Pias1 reverse: 5’-GGACCTGCACTGTGAAGTCA-3’

Pias2 forward: 5’-TGTAGCCAGTGATGCAAGCA-3’

Pias2 reverse: 5’-ACTGAAGTCACACTGGGCAC-3’

Pias3 forward: 5’-TCCCGCTACATGAGTACCCA-3’

Pias3 reverse: 5’-CAGGAAGTGGGAAGGGGTTC-3’

Pias4 forward: 5’-CCCCATCAACCTTACCCACC-3’

Pias4 reverse: 5’-AGGATGCTTCACCCCAATCG-3’

Sae1 forward: 5’-CCTGAGGCCAAGAGAGCAAA-3’

Sae1 reverse: 5’-TGCAGCAGAAAGTAGTCGGG-3’

Sae2 forward: 5’-GGGGCCAAAACAAGCTGAAG-3’

Sae2 reverse: 5’-ACTTCTTGGTACTGGCAGCC-3’

Senp1 forward: 5’-CGAAGTCTTTGCCTCGAAAC-3’

Senp1 reverse: 5’-GCAGGCTTAATGGGAAATGA-3’

Senp2 forward: 5’-CAGCAGTTGAATGGGAGTGA-3’

Senp2 reverse: 5’-AGAGGCATCTGGTGCTGAGT-3’

Senp3 forward: 5’-AGGTGCCATCACCCTGCTG-3’

Senp3 reverse: 5’-GACTTTGGGGTCCACCTTAG-3’

Senp5 forward: 5’-CGAGTTTGTCATGGCTGCTA-3’

Senp5 reverse: 5’-ACGCTCTGACTCTTCGGTGT-3’

Senp6 forward: 5’-GAAGTGGAATGGGAGGTGAA-3’

Senp6 reverse: 5’-TGCAGCACATAGACACCACA-3’

Senp7 forward: 5’-AGCTTGAAGAGTCCGGTGAA-3’

Senp7 reverse: 5’-CTAGCATCTGGCTCCTGACC-3’

Sumo1 forward: 5’-GGACAGGATAGCAGTGAGATACA-3’

Sumo1 reverse: 5’-TCACATCTTCTTCCTCCATTCCC-3’

Sumo2 forward: 5’-AATTTGAAGGTGGCGGGACA-3’

Sumo2 reverse: 5’-TGTTTCGTTGATTGGCTGCC-3’

Sumo3 forward: 5’-GAGCAAGCTGATGAAGGCCT-3’

Sumo3 reverse: 5’-GATCCTCCTGTCTGCTGCTG-3’

Ubc9 forward: 5’-GAAGACAAGGACTGGAGGCC-3’

Ubc9 reverse: 5’-GTGCTCGGACCCTTTTCTCA-3’

Tnni3 forward: 5’- CTCCTCTGCCAACTACCGAG -3’

Tnni3 reverse: 5’- CCCATCCAACTCCAAAGGCT -3’

Myl2 forward: 5’- AAGAAGCGGATAGAAGGCGG -3’

Myl2 reverse: 5’- TTGGACCTGGAGCCTCTTTG -3’

Casq1 forward: 5’- TGGAGGAGCACAGGAGATCA -3’

Casq1 reverse: 5’- TCTCAGTGTTGTCTTGGGCC -3’

MuRF1 forward: 5’- CCAACGACATCTTCCAGGCT -3’

MuRF1 reverse: 5’- TCGTCTTCGTGTTCCTTGCA -3’

Atrogin-1 forward: 5’- CAGCTCACATCCCTGAGTGG -3’

Atrogin-1 reverse: 5’- TAATGTTGCCCACCAGCACA -3’

The following oligos were used for qPCR following chromatin immunoprecipitation:

Atp2a1 promoter forward: 5’-TTCTCAGTTCCAAGCCCACCCC-3’

Atp2a1 promoter reverse: 5’-TCTGTCCCCAAAGATGCGCTCC-3’

Myh1 promoter forward: 5’-GTGAAGGCTGCCAGAAAGAG-3’

Myh1 promoter reverse: 5’-AACACAGAGGACAGGGGATG-3’

The following siRNAs were used for transfection:

siGL2: CGUACGCGGAAUACUUCGA-dTdT

siL3mbtl2: UGACGCCAGUCAUGACUUC-dTdT

### C2C12 cell culture and induction of cachexia

Murine C2C12 myoblast progenitor cells were seeded in T75 flasks at 8 × 10^6^ cells or in 6-well plates at 1 × 10^6^ cells per well in Dulbecco’s modified eagle medium (DMEM) containing 4.5 g/l glucose, 15% fetal bovine serum and 1% Penicillin-Streptomycin. Myogenic differentiation was induced by switching to differentiation medium containing 2% horse serum and the medium was changed daily. The cells were differentiated for 5 days to generate mature myotubes. Cachexia was induced by the addition of 10 ng/ml Tumor necrosis factor α (TNF-α; Roche, Switzerland, cat. no. 11271156001) and 100 ng/ml Interferon γ (INF-γ; Abcam, UK, cat. no. ab9922) for 48 h. For contractility assays, analysis of Ca^2+^ transients and immunofluorescence assays, C2C12 cells were seeded on 10 or 18 mm glass coverslips coated with laminin.

### Human primary cell (HSkM) culture and differentiation

HSkM culture and differentiation was performed according to the manufacturer protocol (A12555, Thermo Fisher Scientific), briefly, on day 2 of differentiation 10 ng/ml TNF-α and 100 ng/ml Interferon γ were added for 48 h.

### Senp activity assay

Differentiated muscle cells were collected with TrypLE and washed thrice with ice-cold PBS. The cells were resuspended in 1 ml SEM buffer (0.25 M sucrose, 1 mM DTT, 20 mM MOPS, pH 7.2) containing 1 mM DTT and protease inhibitor, sonicated on ice for 1 minute total (3 cycles of 20 s ON - 40 s OFF) on a Sonics ultrasonicator at 40% amplitude and spun down for 15 min at 16,000 × *g* and 4 °C. After determination of protein concentration using Bradford reagent, 200 µg protein was added to fresh vials, with 15 mM NEM added to negative controls. One hundred nanograms SUMO2-vinyl sulfonate (VS) was added to each tube and incubated for 15 min at 25 °C on a thermoshaker. The reaction was stopped by addition of 4x ROTI Load 1 and incubated for 7 min at 92 °C. Formation of SENP-SUMO-VS adducts was investigated by western blotting against indicated SENPs.

### Gel electrophoresis and western blotting

Whole cell lysates were collected by washing the cells with PBS and scraping in ROTI Load 1 (Carl Roth, Germany, cat. no. K929) diluted in phosphate-buffered saline (PBS; Gibco, USA, cat. no. 14190136). The samples were incubated for 7 min at 92 °C and equal amounts of protein were loaded on 10% sodium dodecyl sulfate polyacrylamide gels (SDS-PAGE) along with Precision Plus Protein Dual Color Standard (Bio-Rad, USA, cat. no. 1610374) and separated for 70 min at 150 V in a Mini-Protean electrophoresis cell (Bio-Rad, USA, cat. no. 1658006FC). The gels were blotted on 0.45 µm nitrocellulose membranes for 60 min at 10 V in a semi-dry blotter (Bio-Rad, USA, cat. no. 1703940). After blocking for 60 min at room temperature in 4% (w/v) non-fat dry milk (ChemCruz, USA, cat. no. SC-2325) in TBST (150 mM NaCl (Supelco, USA, cat. no. 1.06404), 50 mM Tris (Sigma-Aldrich, USA, cat. no. T1503), pH 7.4, 0.1% (v/v) Tween-20 (Sigma-Aldrich, USA, cat. no. P1379)) primary antibodies were incubated overnight at 4 °C in blocking solution with gentle agitation. After washing in TBST, secondary antibodies were incubated for 60 min at room temperature in blocking solution. The blots were developed with SuperSignal West Dura (Thermo Scientific, USA, cat. no. 34075) and imaged on an ImageQuant LAS4000 System.

### RNA-sequencing

Five hundred nanograms RNA per sample was enriched using ‘NEBNext® Poly(A) mRNA Magnetic Isolation Module’ (E7490L; New England Biolabs) followed by stranded cDNA library generation using ‘NEBNext® Ultra II Directional RNA Library Prep Kit for Illumina’ (E7760L; New England Biolabs) according to the manualE7760 (Version 1.0_02-2017; NEB), except that reactions were reduced to 2/3 of initial volumes. cDNA libraries were barcoded using ‘NEBNext Multiplex Oligos for Illumina – 96 Unique Dual Index Primer Pairs’ (6440S; New England Biolabs), cDNA libraries were amplified with 7 cycles of final pcr and additionally purified using 1.2x ‘Agencourt® AMPure® XP Beads’ (#A63881; Beckman Coulter, Inc.). ‘Bioanalyzer High Sensitivity DNA Assay’ (5067-4626; Agilent Technologies) was used for fragment length distribution and all libraries were quantified with the ‘Qubit® dsDNA HS Assay Kit’ (Q32854; ThermoFisher Scientific). Equal molar amounts of all libraries were pooled for a common sequencing run, denatured with NaOH and was diluted to 1.8 pM according to the Denature and Dilute Libraries Guide (Document # 15048776 v02; Illumina). 1.3 ml of the denatured pool was loaded on an Illumina NextSeq 550 sequencer using a High Output Flowcell (400 M cluster) for single reads (20024906; Illumina). Sequencing was performed with the following settings: Sequence reads 1 and 2 with 38 bases each; Index reads 1 and 2 with 8 bases each. BCL files were converted to FASTQ files using bcl2fastq Conversion Software version v2.20.0.422 (Illumina). Raw data was processed with nfcore/rnaseq (version 1.4.2), using Nextflow. The genome reference and annotation data were taken from GENCODE.org (*Mus musculus*; GRCm38.p6). Normalization and differential expression analysis was performed on the internal Galaxy (version 20.05) instance of the RCU Genomics, Hannover Medical School, Germany using DESeq2 (Galaxy Tool Version 2.11.40.6) at default settings with the following exceptions: “Output normalized counts table” was set to “Yes” and additional filters were disabled (“Turn off outliers replacement”, “Turn off outliers filtering”, and “Turn off independent filtering” set to “Yes”). Cut-offs for identification of differentially expressed genes (DEGs) were set to log_2_ fold change of 0.7 and an adjusted *p*-value of ≤ 0.05. Ontology enrichment analysis was performed with the Database for Annotations, Visualization and Integrated Discovery (DAVID) and metascape. Selected ontology terms were visualized in a circle and chord plot with the GOplot package for R.

### Contractility assay of myotubes

Differentiated myotubes on glass coverslips were placed in a custom-made chamber containing differentiation medium set to 37 °C using a thermistor probe (Green Leaf Scientific) and a temperature controller (TC-II, micro temperature controller, Cell MicroControls). The cells were paced with the Myopacer EP field stimulator (IonOptix, USA) at 1 Hz, 40 V, and a pulse duration of 4 ms in transistor-transistor logic mode (TTL). To check the contractile propensity of the myotubes, videos of at least 15 s duration for each view were recorded on an Olympus IX51 microscope with an optiMOS high speed camera (QImaging, Canada) attached.

### Analysis of Ca^2+^ transients

Differentiated myotubes on glass coverslips were placed in a custom-made perfusion chamber set to 37 °C with the NBD TC2 Bip temperature controller. The cells were perfused in a solution containing 20 mM HEPES, 1.2 mM NaH_2_PO_4_, 0.66 mM MgSO_4_, 117 mM NaCl, 5.7 mM KCl, 5 mM Na-Pyruvate, 1.25 mM CaCl_2_, 10 mM Creatin and 10 mM Glucose set to pH 7.4 at 37 °C. Prior to measurements, the cells were loaded for 60 min with 5 µM Fura-2-AM in serum-free DMEM in the presence of 2.5 mM Probenecid and 0.025% (w/v) Pluronic F-127 and washed twice for 15 min with serum-free DMEM. Ca^2+^ transients were recorded on a MyoCam optical contraction analysis system (IonOptix, USA). The cells were paced with a MyoPacer EP field stimulator at 1 Hz, 25 V, and a pulse duration of 4 ms in TTL mode. Single cell fluorescence measured at 510 nm was recorded after excitation at 340 nm (to detect bound Ca^2+^) and 380 nm (to detect free Ca^2+^). For data analysis the IonWizard software was used. After subtracting the background fluorescence of non-fura-2-treated myotubes the following parameters were calculated: (I) ‘time to peak’ indicates the time between baseline and peak of Ca^2+^ transients, (II) peak height or amplitude compared to baseline in percentage, and (III) time constant (τ) for Ca^2+^ reuptake is estimated by fitting the signal decay with the single exponential decay function.

### Reverse transcription quantitative polymerase chain reaction (RT-qPCR)

C2C12 myotubes from 6-well plates were collected with TrypLE (Gibco, USA, #12604013) and PBS (Gibco, USA, #14190136) and RNA was extracted with the High Pure RNA Isolation Kit (Roche, Switzerland, #11828665001) according to the manual. cDNA was synthesized using the Maxima First Strand cDNA Synthesis Kit (Thermo Scientific, USA, #K1641) according to the manual. qPCR was performed using the PowerUp SYBR Green Master Mix (Applied Biosciences, USA, #A25741) on the QuantStudio 6 Flex System (Thermo Fischer, USA). mRNA expression was quantified using the ∆∆C_T_ method, normalized to *Gapdh* gene expression.

### Immunofluorescence assay and confocal imaging

Differentiated myotubes on glass coverslips were fixated at room temperature for 15 min in 4% (w/v) paraformaldehyde in PBS (Thermo Scientific Chemicals, USA, cat. no. J19943) and washed twice in PBS. The cells were permeabilized for 15 min in 0.2% (v/v) Triton X-100 (Roche, Switzerland, cat. no. 11332481001) and blocked for 30 min in 5% (w/v) bovine serum albumin (Gerbu Biotechnik, Germany, cat. no. 1063) in PBS. Primary antibodies were incubated at room temperature for 60 min in blocking solution, washed twice with PBS, followed by incubation with secondary antibodies as above. Nuclei were stained for 5 min with 4’-6-Diamidino-2-phenylindole (DAPI; Sigma, USA, cat. no. D9542). The coverslips were mounted on glass slides with Fluoroshield (Sigma, USA, cat. no. F6182). Images were collected on an Olympus FV1000 confocal microscope.

### Immunoprecipitation

Immunoprecipitation was performed as in Ref. [54]. In short, myotubes were collected and lysed for 25 min at 4 °C in 50 mM HEPES (pH 7.5), 150 mM NaCl, 1 mM EDTA, 1 mM EGTA, and 0.5% (v/v) Triton X-100 in presence of protease and phosphatase inhibitor. The lysates were centrifuged for 15 min at 16,000 g and 4 °C and the supernatants incubated overnight at 4 °C with indicated antibody or isotype IgG. Antibody complexes were collected with protein G dynabeads for 60 min 4 °C and washed four times on a magnetic rack with 1/4x lysis buffer. Samples were eluted by incubation for 7 min at 92 °C with SDS-PAGE sample buffer.

### Mass spectrometric and data analysis

Proteins were mixed and alkylated by acrylamide and further processed by SDS-PAGE and in gel digested as described. Peptide samples were analysed with a shot-gun approach and data dependent analysis in an LC-MS system (RSLC, Orbitrap Exploris 240, both Thermo Fisher) as described recently [55]. Raw MS data were processed using Max Quant (version 2.0), and Perseus software (version 2.0.6.0) and mouse entries of uniprot DB. Proteins were stated identified by a false discovery rate of 0.01 on protein and peptide level. After MaxQuant analysis, the proteinGroups (PGs) file was loaded into Perseus. All PGs identified by site, reverse, and potential contaminants were removed. For further analysis, only PGs were considered, which were quantified in all three replicates. Intensities were transformed into log_2_ values, normalized by subtracting the column median, and missing values were imputed from normal distribution separately for each column. Following MS analysis, protein groups (PGs) in eluate fractions were filtered over their representative inputs. This resulted in 987 confidently SUMOylated PGs, with 846 and 812 PGs in control and cachectic eluates, respectively (Fig. 3A). Please note, the common PGs between these two groups were not taken into account. That is the reason why 846 + 812 is not equal to 1658 but 987. To quantify changes in SUMOylation, filtered control eluate values were subtracted from their respective cachectic value. A change Log_2_ of ≥1 indicates a PG with increased SUMOylation in CIC, while a change Log_2_ of ≤ −1 indicates a PG with decreased SUMOylation in CIC. Significance was calculated by two-way ANOVA with a cut-off of *p* ≤ 0.05.

### Chromatin immunoprecipitation

Chromatin immunoprecipitation (ChIP) was performed as in Ref. [54] with minor modifications. Briefly, chromatin was sheared in a Covaris M220 focused sonicator for 12 min set to 10% duty factor, 75 W peak incident power, 200 cycles per burst, at 7 °C. Sheared chromatin was used for immunoprecipitation assays (using specific antibodies as described in the figures). The DNA from input and immunoprecipitated chromatin was isolated by incubating Protein G dynabeads (that captured immunoprecipitated chromatin-antibody complex) with 100 µl 10% (w/v) Chelex-100, for 10 min at 1200 rpm and 95 °C in a thermomixer. The beads were spun down and the reversed cross-linked DNA was used for qPCR. The diluted input was adjusted to 100% by subtracting 5.05 of the raw *C*_p_ values (Log_2_ (33.3) = 5.05). The percentage of input was calculated by 100 × 2^(C_p_(adjusted input) – C_p_(ChIP eluates)).

### siRNA transfection and plasmid transfection

Transfection of siRNA was performed on day 2 of differentiation using the Lipofectamine RNAiMAX according to manufacturer’s instructions. In short, C2C12 cells were transfected on day 2 of differentiation with 100 pmol of the indicated siRNA using Lipofectamine RNAiMAX according to the manual. The cells were collected on day 5 of differentiation.1 µg of plasmid was transfected on day 2 of differentiation using 7.5 µl TransIT reagent (Mirus) in 300 µl OptiMEM.

### Mice tissue lysate preparation

We used C-26 (murine colon adenocarcinoma) mice model in this study. Fourteen days post subcutaneous inoculation of C26 cells, the mice (Balb/c strain, 12 weeks old) displayed significant body weight loss of ~18%. EDL muscles from 3 cachectic mice and 3 control (injected with PBS) were isolated. Protein lysate (from ~18 mg of muscle tissue) was prepared by using modified RIPA buffer (50 mM Tris-HCl, pH 7.6, 150 mM NaCl, 0.5% sodium deoxycholate, 0.1% SDS, 1% IGEPAL CA-630). The buffer was freshly supplemented with protease inhibitor, phosphatase inhibitors and 10 mM n-ethylmaleimide (NEM). Approximately 40 µg of protein samples from different mice and different cohorts was loaded in the SDS-PAGE to monitor various muscle-related protein expression as described in Fig. S1D.

### Statistical analysis

Figures were prepared with the tidyverse software for R. Details of statistical analysis are mentioned in each figure legends.

## Supplementary information

Supplementary Figures

Supplemental Video S1

Supplemental Table (Xlsx)

Original blots

## Data Availability

The data supporting the findings of this study are available from the corresponding author (Nayak.Arnab@mh-hannover.de, nayakarnab@yahoo.com) upon request.

## References

[1] von Haehling S, Anker MS, Anker SD. Prevalence and clinical impact of cachexia in chronic illness in Europe, USA, and Japan: facts and numbers update 2016. J Cachexia Sarcopenia Muscle. 2016;7:507–9. 10.1002/jcsm.12167. 27891294 PMC5114624

[2] Gould DW, Lahart I, Carmichael AR, Koutedakis Y, Metsios GS. Cancer cachexia prevention via physical exercise: molecular mechanisms. J Cachexia Sarcopenia Muscle. 2013;4:111–24. 10.1007/s13539-012-0096-0. 23239116 PMC3684702

[3] Murphy BT, Mackrill JJ, O’Halloran KD. Impact of cancer cachexia on respiratory muscle function and the therapeutic potential of exercise. J Physiol. 2022. 10.1113/JP283569 PMC10091733 36251564

[4] Wang G, Biswas AK, Ma W, Kandpal M, Coker C, Grandgenett PM, et al. Metastatic cancers promote cachexia through ZIP14 upregulation in skeletal muscle. Nat Med. 2018;24:770–81. 10.1038/s41591-018-0054-2. 29875463 PMC6015555

[5] Biswas AK, Acharyya S. Understanding cachexia in the context of metastatic progression. Nat Rev Cancer. 2020;20:274–84. 10.1038/s41568-020-0251-4. 32235902

[6] Adams V, Gussen V, Zozulya S, Cruz A, Moriscot A, Linke A, et al. Small-molecule chemical knockdown of MuRF1 in melanoma bearing mice attenuates tumor cachexia associated myopathy. Cells. 2020;9. 10.3390/cells9102272. PMC7600862 33050629

[7] Bowen TS, Adams V, Werner S, Fischer T, Vinke P, Brogger MN, et al. Small-molecule inhibition of MuRF1 attenuates skeletal muscle atrophy and dysfunction in cardiac cachexia. J Cachexia Sarcopenia Muscle. 2017;8:939–53. 10.1002/jcsm.12233. 28887874 PMC5700443

[8] Groarke JD, Crawford J, Collins SM, Lubaczewski S, Roeland EJ, Naito T, et al. Ponsegromab for the treatment of cancer cachexia. N Engl J Med. 2024;391:2291–303. 10.1056/NEJMoa2409515. 39282907

[9] Flotho A, Melchior F. Sumoylation: a regulatory protein modification in health and disease. Annu Rev Biochem. 2013;82:357–85. 10.1146/annurev-biochem-061909-093311. 23746258

[10] Wang Y, Dasso M. SUMOylation and deSUMOylation at a glance. J Cell Sci. 2009;122:4249–52. 10.1242/jcs.050542. 19923268 PMC2779127

[11] Yeh ET. SUMOylation and De-SUMOylation: wrestling with life’s processes. J Biol Chem. 2009;284:8223–7. 10.1074/jbc.R800050200. 19008217 PMC2659178

[12] Chang HM, Yeh ETH. SUMO: from bench to bedside. Physiol Rev. 2020;100:1599–619. 10.1152/physrev.00025.2019. 32666886 PMC7717128

[13] Cubenas-Potts C, Matunis MJ. SUMO: a multifaceted modifier of chromatin structure and function. Dev Cell. 2013;24:1–12. 10.1016/j.devcel.2012.11.020. 23328396 PMC3555686

[14] Hay RT. SUMO: a history of modification. Mol Cell. 2005;18:1–12. 10.1016/j.molcel.2005.03.012. 15808504

[15] Nayak A, Muller S. SUMO-specific proteases/isopeptidases: SENPs and beyond. Genome Biol. 2014;15:422. 10.1186/s13059-014-0422-2. 25315341 PMC4281951

[16] Vertegaal ACO. Signalling mechanisms and cellular functions of SUMO. Nat Rev Mol Cell Biol. 2022;23:715–31. 10.1038/s41580-022-00500-y. 35750927

[17] Celen AB, Sahin U. Sumoylation on its 25th anniversary: mechanisms, pathology, and emerging concepts. FEBS J. 2020;287:3110–40. 10.1111/febs.15319. 32255256

[18] Pichler A, Fatouros C, Lee H, Eisenhardt N. SUMO conjugation - a mechanistic view. Biomol Concepts. 2017;8:13–36. 10.1515/bmc-2016-0030. 28284030

[19] Kunz K, Wagner K, Mendler L, Holper S, Dehne N, Muller S. SUMO signaling by hypoxic inactivation of SUMO-specific isopeptidases. Cell Rep. 2016;16:3075–86. 10.1016/j.celrep.2016.08.031. 27626674

[20] Liebelt F, Sebastian RM, Moore CL, Mulder MPC, Ovaa H, Shoulders MD, et al. SUMOylation and the HSF1-regulated chaperone network converge to promote proteostasis in response to heat shock. Cell Rep. 2019;26:236–249. e4. 10.1016/j.celrep.2018.12.027. 30605679 PMC6316133

[21] Antunes D, Padrao AI, Maciel E, Santinha D, Oliveira P, Vitorino R, et al. Molecular insights into mitochondrial dysfunction in cancer-related muscle wasting. Biochim Biophys Acta. 2014;1841:896–905. 10.1016/j.bbalip.2014.03.004. 24657703

[22] Zhang Z, Tan S, Li S, Cheng Y, Wang J, Liu H, et al. Mitophagy-mediated inflammation and oxidative stress contribute to muscle wasting in cancer cachexia. J Clin Biochem Nutr. 2023;73:34–42. 10.3164/jcbn.23-1. 37534096 PMC10390805

[23] Soshnikova N, Duboule D. Epigenetic temporal control of mouse Hox genes in vivo. Science. 2009;324:1320–3. 10.1126/science.1171468. 19498168

[24] Gerasimova TI, Corces VG. Polycomb and trithorax group proteins mediate the function of a chromatin insulator. Cell. 1998;92:511–21. 10.1016/s0092-8674(00)80944-7. 9491892

[25] Schuettengruber B, Bourbon HM, Di Croce L, Cavalli G. Genome regulation by polycomb and trithorax: 70 years and counting. Cell. 2017;171:34–57. 10.1016/j.cell.2017.08.002. 28938122

[26] Ringrose L, Paro R. Epigenetic regulation of cellular memory by the Polycomb and Trithorax group proteins. Annu Rev Genet. 2004;38:413–43. 10.1146/annurev.genet.38.072902.091907. 15568982

[27] Shilatifard A. The COMPASS family of histone H3K4 methylases: mechanisms of regulation in development and disease pathogenesis. Annu Rev Biochem. 2012;81:65–95. 10.1146/annurev-biochem-051710-134100. 22663077 PMC4010150

[28] Ernst P, Vakoc CR. WRAD: enabler of the SET1-family of H3K4 methyltransferases. Brief Funct Genom. 2012;11:217–26. 10.1093/bfgp/els017. PMC3388306 22652693

[29] Bonasio R, Lecona E, Reinberg D. MBT domain proteins in development and disease. Semin Cell Dev Biol. 2010;21:221–30. 10.1016/j.semcdb.2009.09.010. 19778625 PMC3772645

[30] Aranda S, Mas G, Di Croce L. Regulation of gene transcription by Polycomb proteins. Sci Adv. 2015;1:e1500737. 10.1126/sciadv.1500737. 26665172 PMC4672759

[31] Guttridge DC, Mayo MW, Madrid LV, Wang CY, Baldwin AS. NF-kappaB-induced loss of MyoD messenger RNA: possible role in muscle decay and cachexia. Science. 2000;289:2363–6. 10.1126/science.289.5488.2363. 11009425

[32] Gand LV, Lanzuolo C, Li M, Rosti V, Weber N, Lu D, et al. Calcium handling machinery and sarcomere assembly are impaired through multipronged mechanisms in cancer cytokine-induced cachexia. J Cachexia Sarcopenia Muscle. 2025;16:e13776. 10.1002/jcsm.13776. 40183240 PMC11969248

[33] Talbert EE, Metzger GA, He WA, Guttridge DC. Modeling human cancer cachexia in colon 26 tumor-bearing adult mice. J Cachexia Sarcopenia Muscle. 2014;5:321–8. 10.1007/s13539-014-0141-2. 24668658 PMC4248405

[34] Bonetto A, Rupert JE, Barreto R, Zimmers TA. The colon-26 carcinoma tumor-bearing mouse as a model for the study of cancer cachexia. J Vis Exp. 2016;54893. 10.3791/54893. PMC5226332 27929469

[35] Barysch SV, Dittner C, Flotho A, Becker J, Melchior F. Identification and analysis of endogenous SUMO1 and SUMO2/3 targets in mammalian cells and tissues using monoclonal antibodies. Nat Protoc. 2014;9:896–909. 10.1038/nprot.2014.053. 24651501

[36] Namuduri AV, Heras G, Mi J, Cacciani N, Hornaeus K, Konzer A, et al. A proteomic approach to identify alterations in the small ubiquitin-like modifier (SUMO) network during controlled mechanical ventilation in rat diaphragm muscle. Mol Cell Proteomics. 2017;16:1081–97. 10.1074/mcp.M116.066159. 28373296 PMC5461539

[37] Zhou Y, Zhou B, Pache L, Chang M, Khodabakhshi AH, Tanaseichuk O, et al. Metascape provides a biologist-oriented resource for the analysis of systems-level datasets. Nat Commun. 2019;10:1523. 10.1038/s41467-019-09234-6. 30944313 PMC6447622

[38] Stielow C, Stielow B, Finkernagel F, Scharfe M, Jarek M, Suske G. SUMOylation of the polycomb group protein L3MBTL2 facilitates repression of its target genes. Nucleic Acids Res. 2014;42:3044–58. 10.1093/nar/gkt1317. 24369422 PMC3950706

[39] Calderon JC, Bolanos P, Caputo C. The excitation-contraction coupling mechanism in skeletal muscle. Biophys Rev. 2014;6:133–60. 10.1007/s12551-013-0135-x. 28509964 PMC5425715

[40] Trojer P, Cao AR, Gao Z, Li Y, Zhang J, Xu X, et al. L3MBTL2 protein acts in concert with PcG protein-mediated monoubiquitination of H2A to establish a repressive chromatin structure. Mol Cell. 2011;42:438–50. 10.1016/j.molcel.2011.04.004. 21596310 PMC3142354

[41] Rampalli S, Li L, Mak E, Ge K, Brand M, Tapscott SJ, et al. p38 MAPK signaling regulates recruitment of Ash2L-containing methyltransferase complexes to specific genes during differentiation. Nat Struct Mol Biol. 2007;14:1150–6. 10.1038/nsmb1316. 18026121 PMC4152845

[42] Zhou H, Feng W, Yu J, Shafiq TA, Paulo JA, Zhang J, et al. SENP3 and USP7 regulate Polycomb-rixosome interactions and silencing functions. Cell Rep. 2023;42:112339. 10.1016/j.celrep.2023.112339. 37014752 PMC10777863

[43] Nayak A, Viale-Bouroncle S, Morsczeck C, Muller S. The SUMO-specific isopeptidase SENP3 regulates MLL1/MLL2 methyltransferase complexes and controls osteogenic differentiation. Mol Cell. 2014;55:47–58. 10.1016/j.molcel.2014.05.011. 24930734

[44] Delfinis LJ, Bellissimo CA, Gandhi S, DiBenedetto SN, Garibotti MC, Thuhan AK, et al. Muscle weakness precedes atrophy during cancer cachexia and is linked to muscle-specific mitochondrial stress. JCI Insight. 2022;7:e155147. 10.1172/jci.insight.155147. 36346680 PMC9869968

[45] Roberts BM, Ahn B, Smuder AJ, Al-Rajhi M, Gill LC, Beharry AW, et al. Diaphragm and ventilatory dysfunction during cancer cachexia. FASEB J. 2013;27:2600–10. 10.1096/fj.12-222844. 23515443 PMC3688752

[46] Argilés JM, López-Soriano FJ, Stemmler B, Busquets S. Cancer-associated cachexia - understanding the tumour macroenvironment and microenvironment to improve management. Nat Rev Clin Oncol. 2023;20:250–64. 10.1038/s41571-023-00734-5. 36806788

[47] Hain BA, Xu H, Waning DL. Loss of REDD1 prevents chemotherapy-induced muscle atrophy and weakness in mice. J Cachexia Sarcopenia Muscle. 2021;12:1597–612. 10.1002/jcsm.12795. 34664403 PMC8718092

[48] Law ML, Metzger JM. Cardiac myocyte intrinsic contractility and calcium handling deficits underlie heart organ dysfunction in murine cancer cachexia. Sci Rep. 2021;11:23627. 10.1038/s41598-021-02688-z. 34880268 PMC8655071

[49] Sartori R, Hagg A, Zampieri S, Armani A, Winbanks CE, Viana LR, et al. Perturbed BMP signaling and denervation promote muscle wasting in cancer cachexia. Sci Transl Med. 2021;13:eaay9592. 10.1126/scitranslmed.aay9592. 34349036

[50] Acharyya S, Ladner KJ, Nelsen LL, Damrauer J, Reiser PJ, Swoap S, et al. Cancer cachexia is regulated by selective targeting of skeletal muscle gene products. J Clin Investig. 2004;114:370–8. 10.1172/JCI20174. 15286803 PMC484974

[51] Gao S, Zhang G, Zhang Z, Zhu JZ, Li L, Zhou Y, et al. UBR2 targets myosin heavy chain IIb and IIx for degradation: molecular mechanism essential for cancer-induced muscle wasting. Proc Natl Acad Sci USA. 2022;119:e2200215119. 10.1073/pnas.2200215119. 36252004 PMC9618047

[52] Banduseela V, Ochala J, Lamberg K, Kalimo H, Larsson L. Muscle paralysis and myosin loss in a patient with cancer cachexia. Acta Myol. 2007;26:136–44. 18646562 PMC2949304

[53] Khan B, Lanzuolo C, Rosti V, Santarelli P, Pich A, Kraft T, et al. Sorafenib induces cachexia by impeding transcriptional signaling of the SET1/MLL complex on muscle-specific genes. iScience. 2024;27:110913. 10.1016/j.isci.2024.110913. 39386761 PMC11462028

[54] Nayak A, Lopez-Davila AJ, Kefalakes E, Holler T, Kraft T, Amrute-Nayak M. Regulation of SETD7 methyltransferase by SENP3 is crucial for sarcomere organization and cachexia. Cell Rep. 2019;27:2725–2736. e4. 10.1016/j.celrep.2019.04.107. 31141694

[55] Stieglitz F, Gerhard R, Hönig R, Giehl K, Pich A. TcdB of clostridioides difficile mediates RAS-dependent necrosis in epithelial cells. Int J Mol Sci. 2022;23:4258 10.3390/ijms23084258. 35457076 PMC9024770

