# Supplementary material for: Reorientation of the SUMOylation landscape and altered L3mbtl2-mediated transcriptional activity in cancer-associated muscle contractile dysfunction
Source: Cell Death Discov. 2026 Sep 9;12:360. doi: 10.1038/s41420-026-03337-y (PMC13558696; doi:10.1038/s41420-026-03337-y)
Supplement: Supplementary file 1 [file 41420_2026_3337_MOESM1_ESM.pdf]

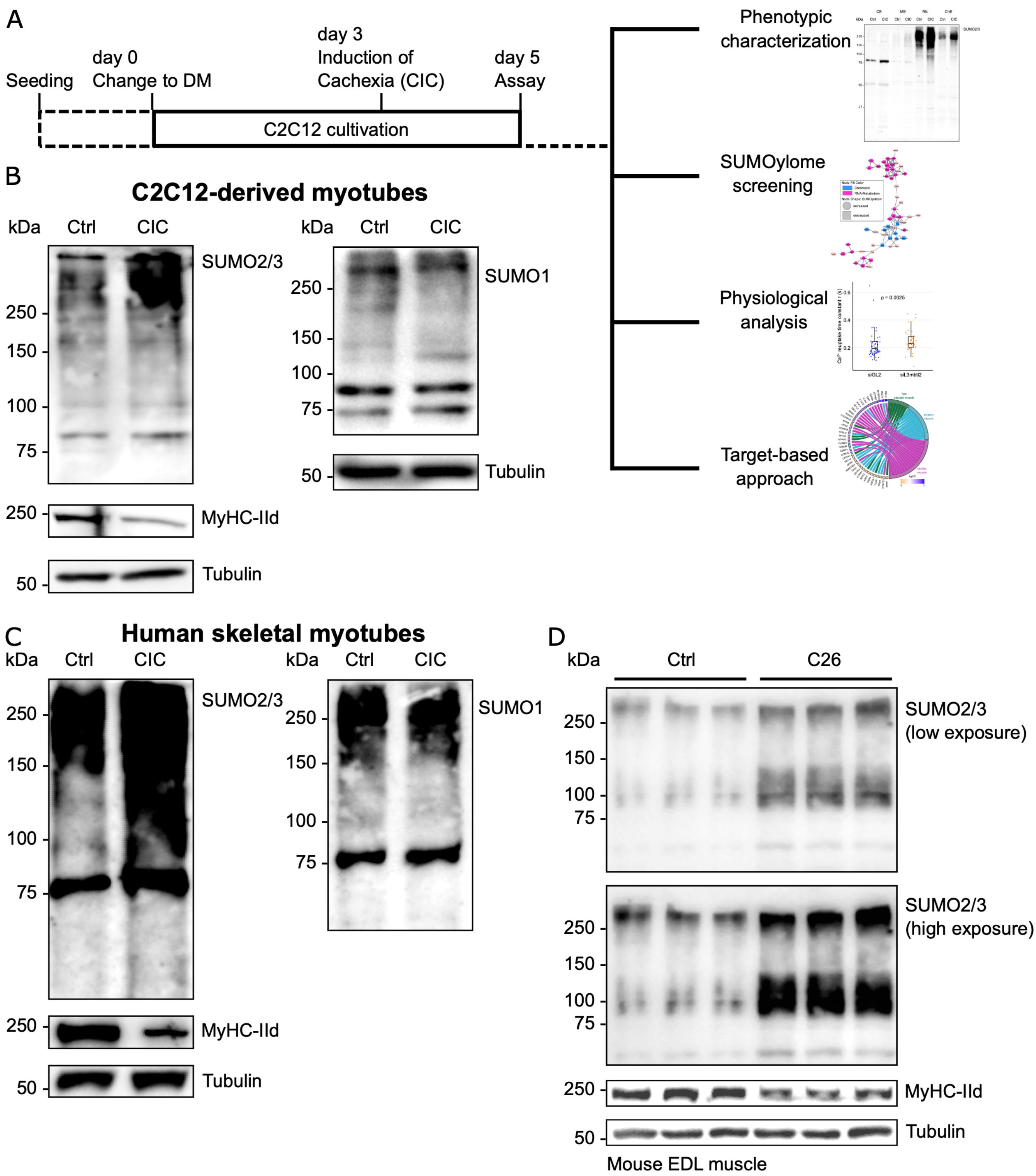

Figure S1 (related to figure 1): A) Scheme illustrating mouse C2C12 progenitor myoblast differentiation into mature muscle cells (myotubes), induction of CIC and the experimental setup. B) Immunoblots of control and cachectic lysates probed with indicated antibodies (N = 3 biological replicates). C) Same as in B), but lysates are derived from primary human skeletal myotubes. D) EDL muscle lysates from 3 control mice and 3 C-26 tumor beaning cancer cachectic mice were probed in western blots with indicated antibodies.

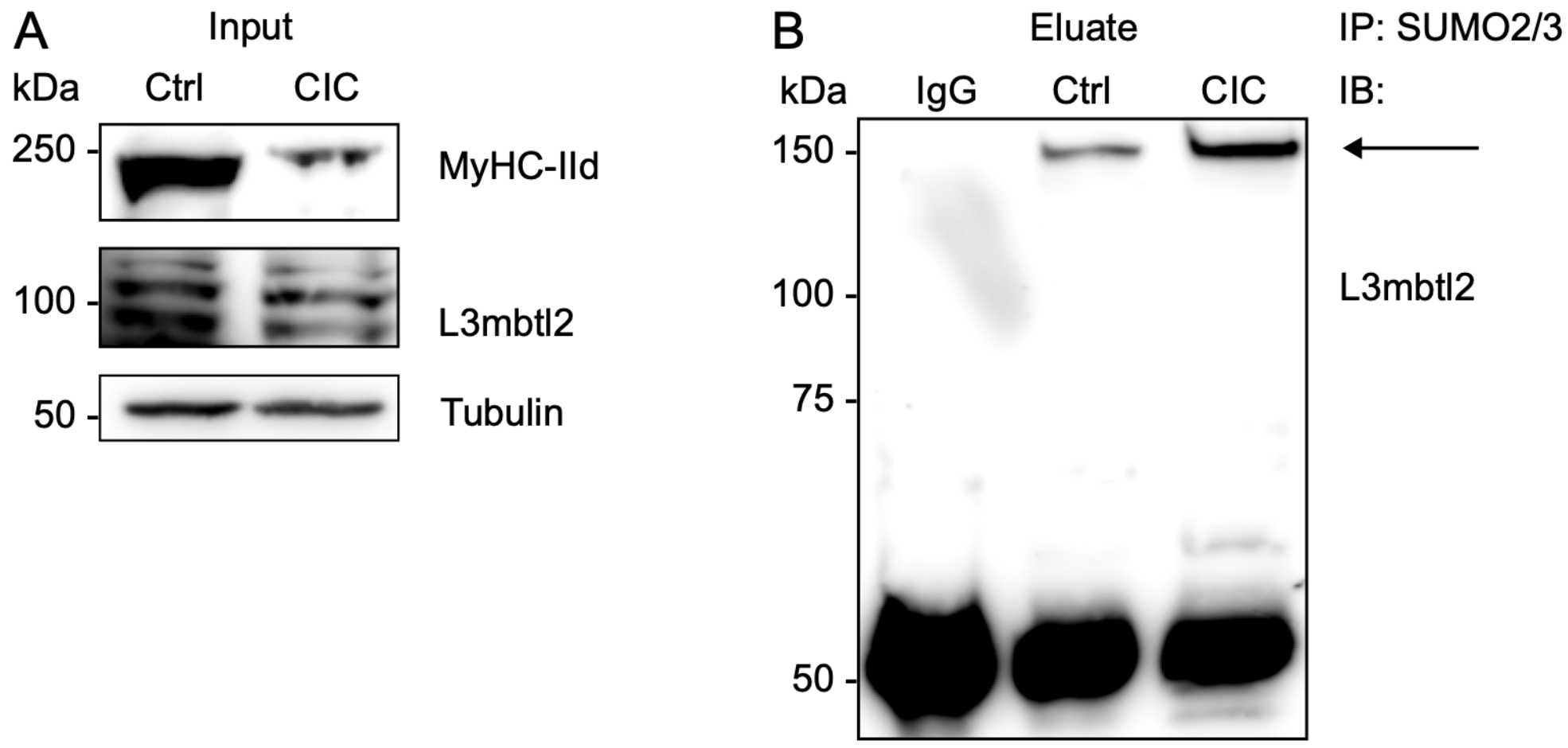

Figure S2 (related to Figure 2): A) Immunoblot of Ctrl and CIC cell lysates (input samples) probed for MyHC-IId, L3mbtl2, and tubulin as a loading control. B) Eluate samples after immunoprecipitation using a SUMO2/3-specific antibody probed against L3mbtl2. Arrow shows higher molecular weight SUMO-modified species of L3mbtl2.

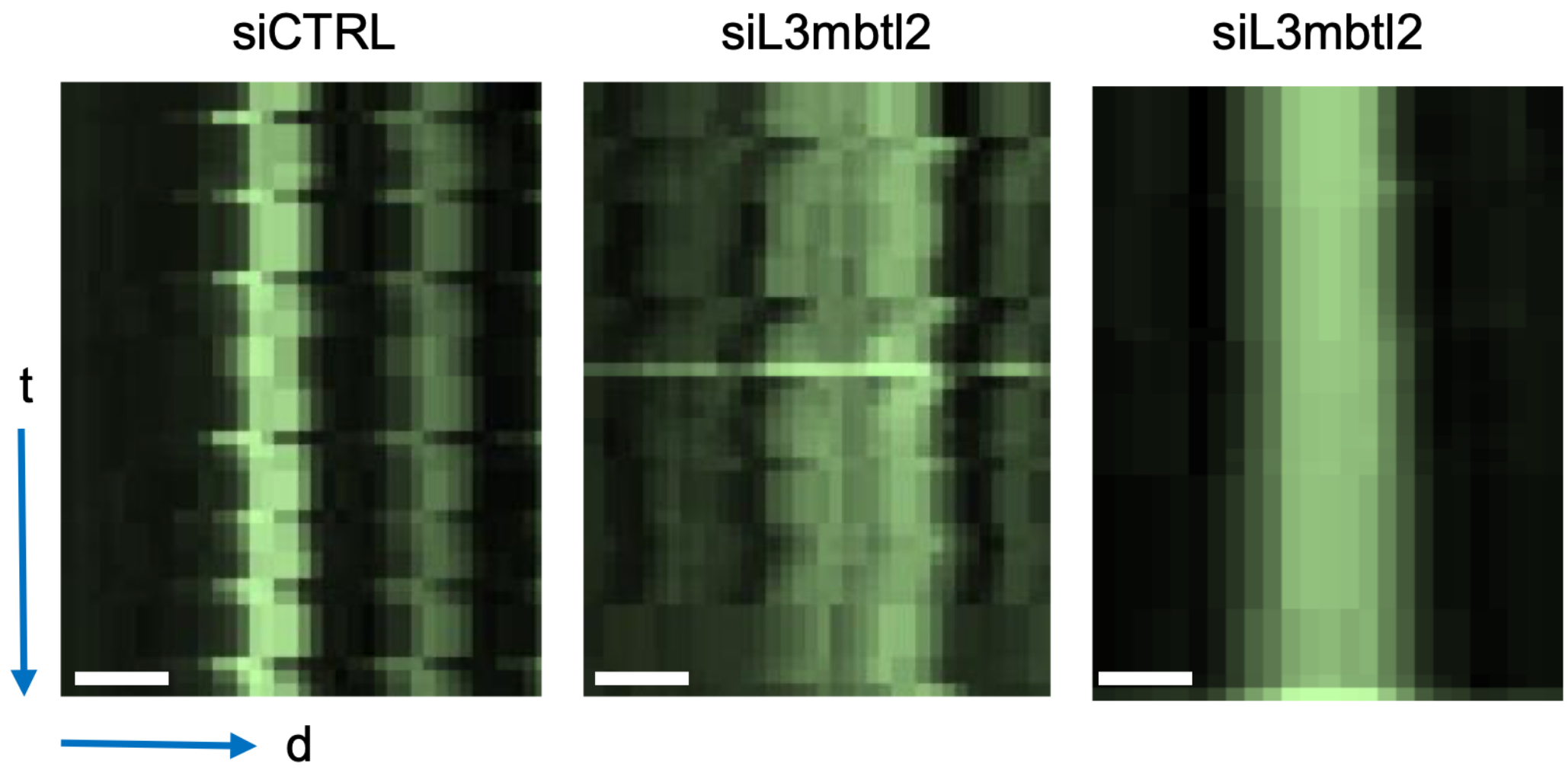

Figure S3 (related to Figure 3): (A) Myotube's contraction ability was assessed using pacing experiment. During contraction, movement of intracellular structures or organelles within myotubes was observed (Supplementary movie S1). Kymographs (time, t vs. distance, d plot) were generated by drawing a line along the length of the myotube's. In a kymograph, each selected particle appears as a line, with line thickness corresponding to the particle size. For each image stack, changes in intensity along the plotted line were monitored over time. Regular horizontal shifts in intensity, indicative of contraction, were observed in control siRNA cells (left panel). In contrast, non-contractile L3mbtl2 knockdown cells showed no periodic intensity shifts in the kymograph (right panel). The contractile cohorts of L3mbtl2 knockdown cells (middle panel) exhibited less discrete and less regular (contraction) movement over time compared to control cells. Scale bar corresponds to 5 μm.

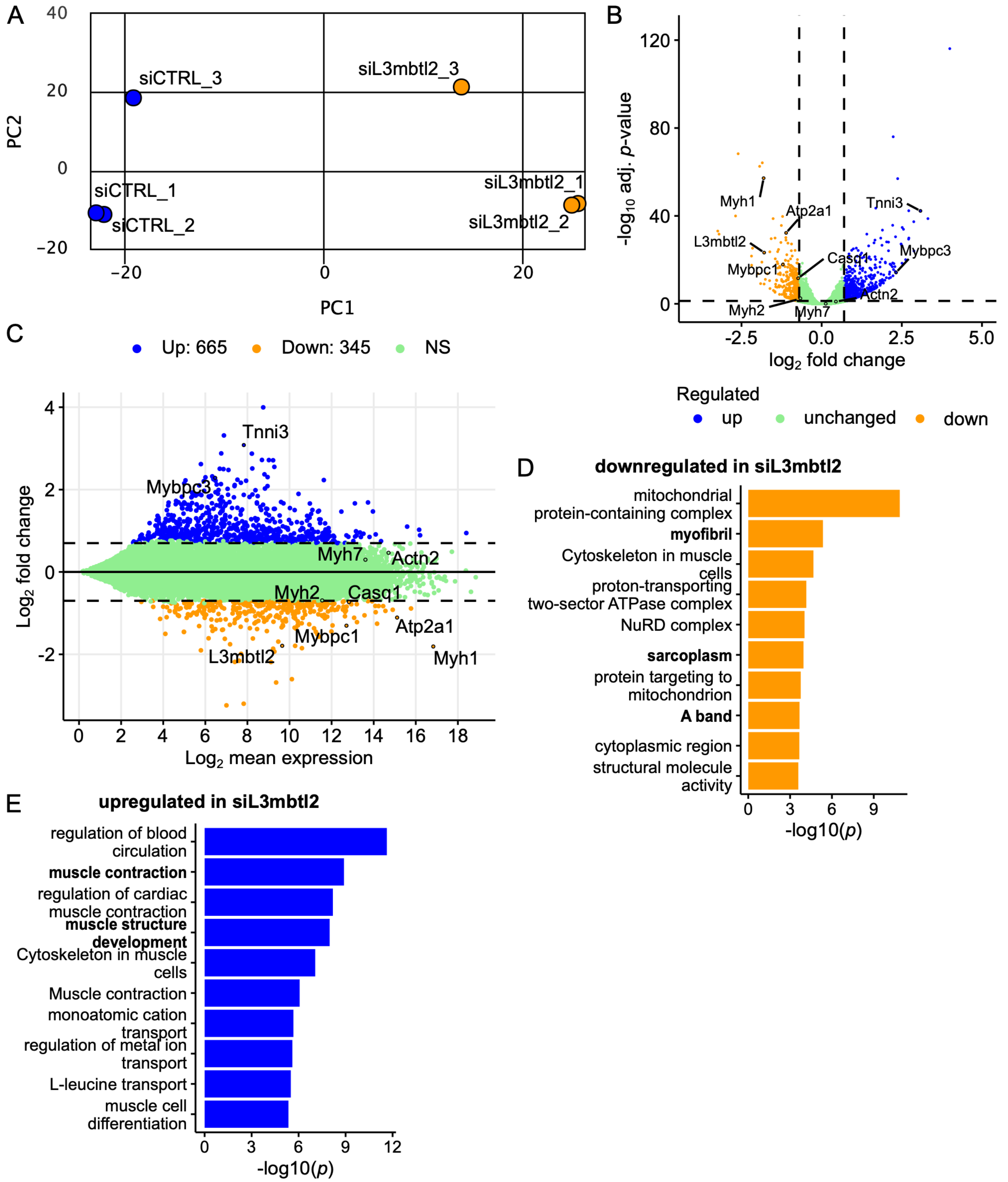

Figure S4 (related to Figure 3): A) Pearson correlation analysis (PCA) plot shows clustering of siCTRL samples is distinct from siL3mbtl2 samples. B) Volcano plot shows $\log_2$ fold change of transcript levels and $-\log_{10}$ *p* adj. value on x and y axis, respectively. N= 3 biological replicates. C) MA plot shows the $\log_2$ fold change in relation to the $\log_2$ mean expression of each transcript, NS: not significant. D-E) MetaScape analysis reveals enriched gene ontology terms in genes D) downregulated and E) upregulated by knockdown of *L3mbtl2*.

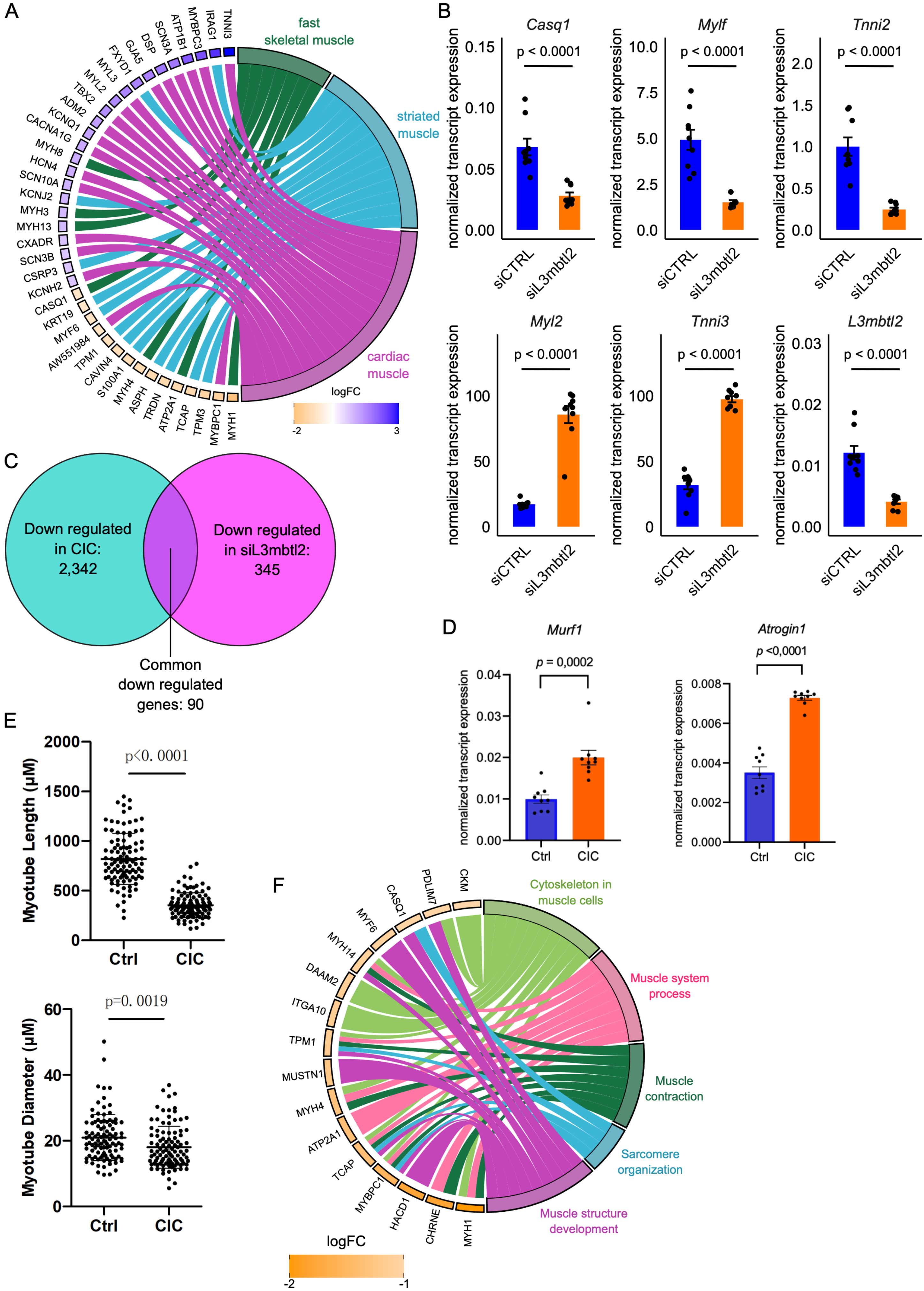

A
fast skeletal muscle
striated muscle
cardiac muscle
logFC
-2
3
TNNI3
IRAG1
MYBPC3
ATP1B1
SCN3A
DSP
GJA5
FXYD1
MYL3
MYL2
TBX2
ADM2
KCNQ1
CACNA1G
MYH8
HCN4
SCN10A
KCNJ2
MYH3
MYH13
CXADR
SCN3B
CSRP3
KCNH2
CASQ1
KRT19
MYF6
AW551984
TPM1
CAVIN4
S100A1
MYH4
ASPH
TRDN
ATP2A1
TCAP
TPM3
MYBPC1
MYH1
B
Casq1
Mylf
Tnni2
Myl2
Tnni3
L3mbtl2
normalized transcript expression
p < 0.0001
siCTRL
siL3mbtl2
C
Down regulated in CIC: 2,342
Down regulated in siL3mbtl2: 345
Common down regulated genes: 90
D
Murf1
Atrogin1
p = 0,0002
p <0,0001
normalized transcript expression
Ctrl
CIC
E
Myotube Length (μM)
p<0. 0001
Myotube Diameter (μM)
p=0. 0019
Ctrl
CIC
F
Cytoskeleton in muscle cells
Muscle system process
Muscle contraction
Sarcomere organization
Muscle structure development
CKM
PDLIM7
CASQ1
MYF6
MYH14
DAAM2
ITGA10
TPM1
MUSTN1
MYH4
ATP2A1
TCAP
MYBPC1
HACD1
CHRNE
MYH1
logFC
-2
-1

Figure S5 (related to Figure 3): A) Chord plot analysis of muscle-specific genes regulated by *L3mbtl2* depletion shows major differences between fast skeletal- and cardiac-specific genes. (B) Similar to Figure 1B except, *Casq1, Tnni2, Mylf, Myl2, Tnni3* and *L3mbtl2* gene expression were followed upon L3mbtl2 siRNA. Data presented as mean of three biological replicate and total of 9 technical replicates, statistical significance tested by an unpaired two-sided t-test. (C) Venn diagram shows compilation of two separate RNAseq datasets, i.e. from *L3mbtl2*-depleted myotubes and CIC myotubes. (D) Similar to Figure 1B except, *Murf1* and *Atrogin-1* gene expression in CIC were followed. Data represents mean of three biological replicate and total of 9 technical replicates, statistical significance tested by an unpaired two-sided t-test. (E) Graph shows myotubes' length and diameter in CIC. Data presents 102 cells (in each condition) from three different experiment. Data represents mean (± SD) of three biological experiments with an unpaired two-sided t-test. Myotube length was decreased by ~57% and diameter by ~14% in CIC. Myotube length and thickness was measured for control and drug CIC cells using ImageJ analysis tools. The images were originated from confocal microscopy with alpha-actinin and DAPI co-staining. The images were captured using a 10X objective. (F) Chord plot showing genes and their relatedness to muscle-specific terms enriched among those transcripts downregulated in L3mbtl2 depleted condition.

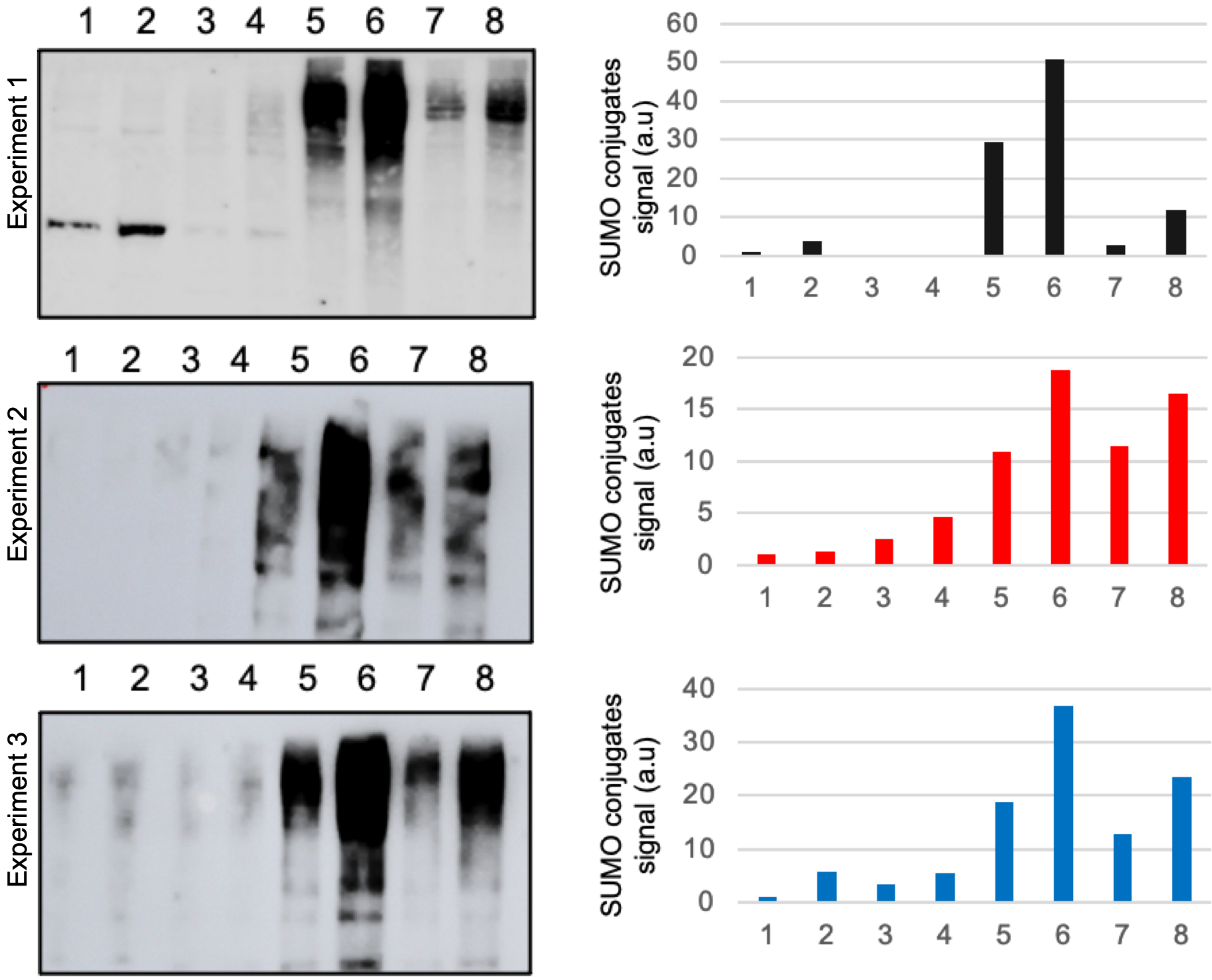

1. Cytosolic extract (CE) Ctrl
2. Cytosolic extract (CE) CIC
3. Membrane extract (ME) Ctrl
4. Membrane extract (ME) CIC
5. Nuclear soluble extract (NE) Ctrl
6. Nuclear soluble extract (NE) CIC
7. Chromatin-bound extract (ChE) Ctrl
8. Chromatin-bound extract (ChE) CIC

Figure S6 (related to Figure 1A): ImageJ analysis shows relative amount of SUMO2/3 signals in different subcellular fractions from Ctrl and CIC conditions (three biological experiments). Cytosolic fraction of control cell value was set as 1 (for each experiment).

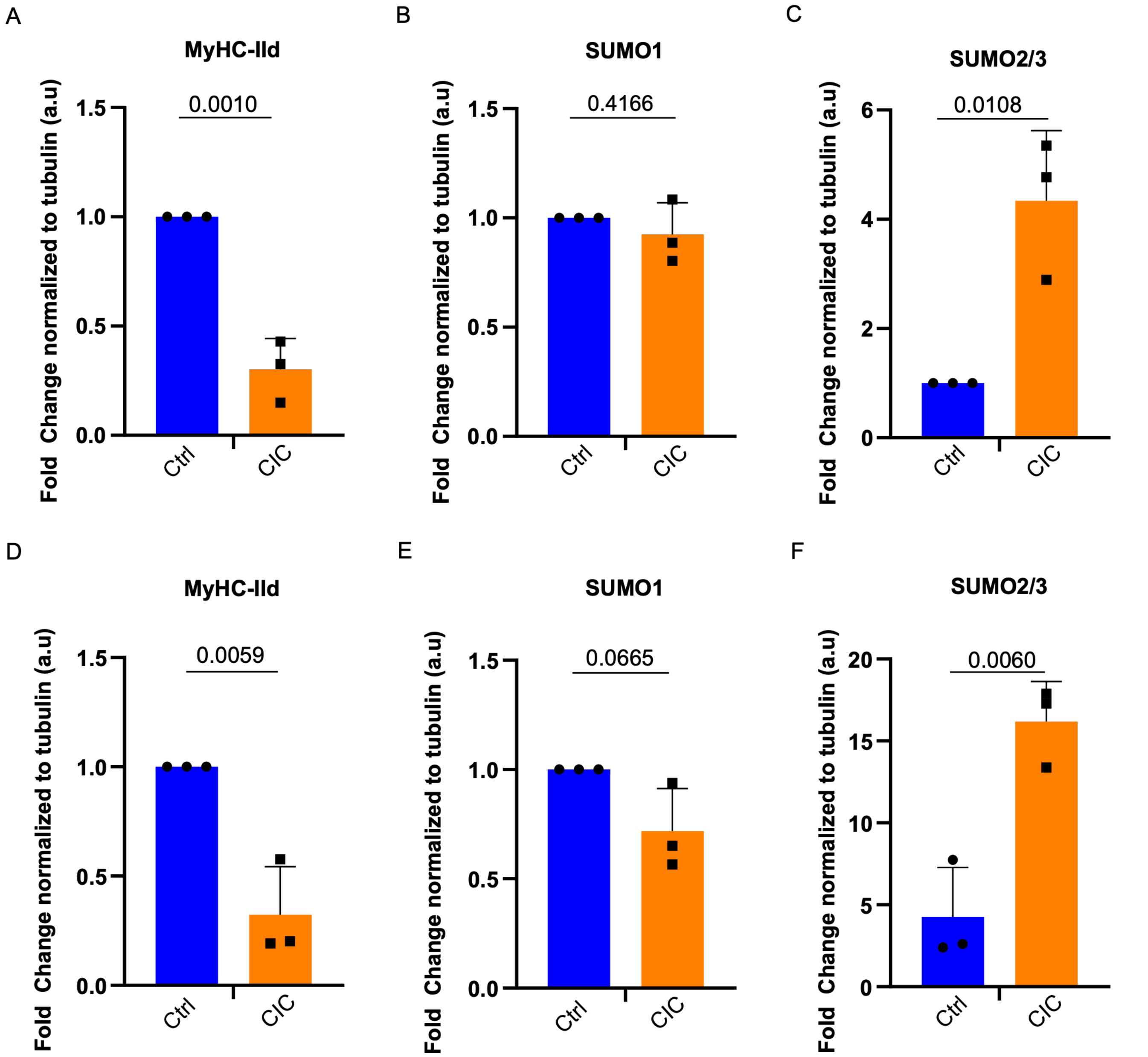

Figure S7 (related to Figure S1B and S1C): ImageJ analysis shows relative amount indicated protein signals from three biological experiments. (A-C) Related to Figure S1B. (D-F) Related to Figure S1C.

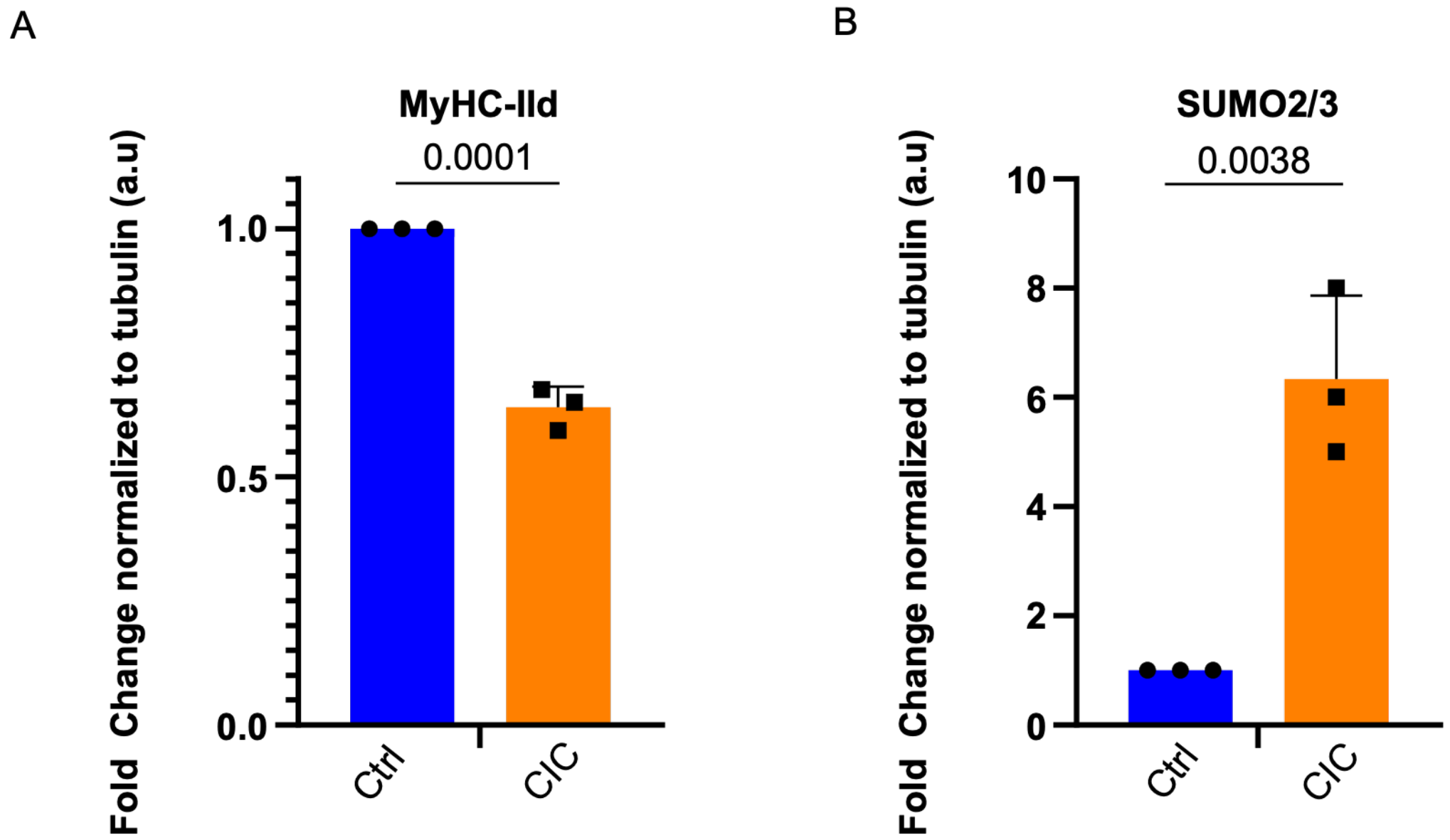

Figure S8 (related to Figure S1D): ImageJ analysis shows relative amount of indicated protein signals (nornalized against tubulin) from EDL muscle tissues isolated from three C26 EDL mice and three Ctrl littermates. Data represent mean of at least three biological experiments (± SD), p-values (unpaired t-test) are provided in the figure.

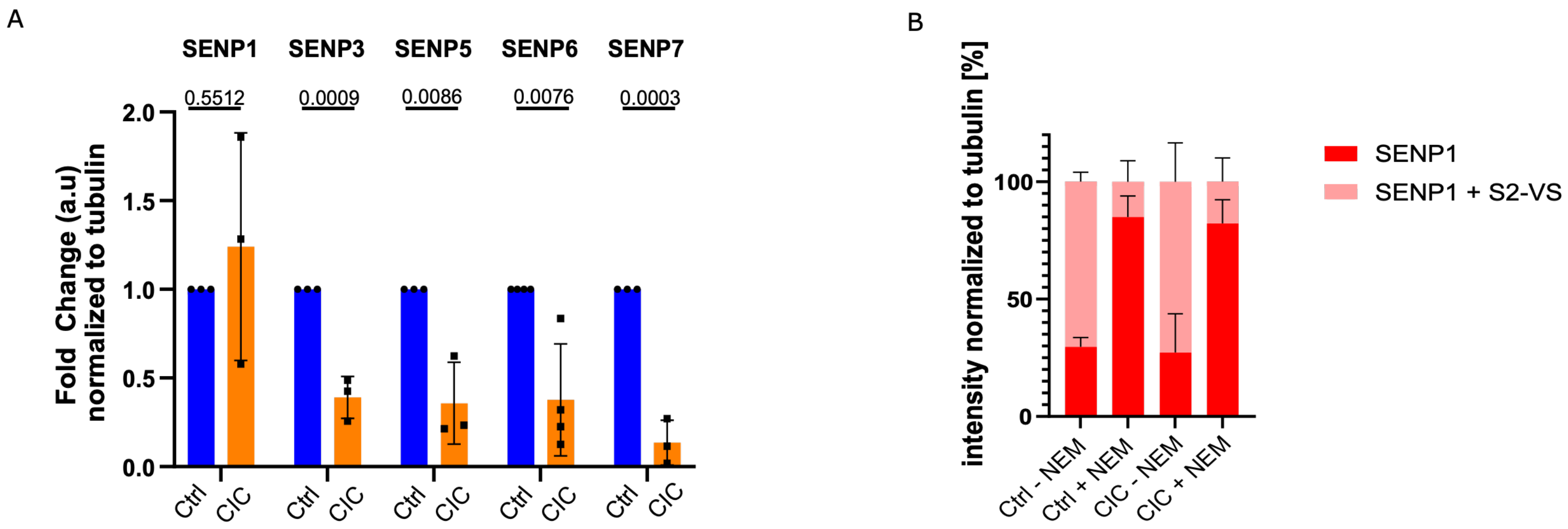

Figure S9 (related to Figure 1E): (A) Analysis of Fig1E at least from three biological replicates. Senp signals were nornalized against tubulin and control value was set as 1. Data represent mean of at least three biological experiments (± SD), p-values (unpaired t-test) are provided in the figure. No significant change was observed for SENP1 expression in CIC. In CIC condition, SENP3, SENP5, SENP6 and SENP7 were reduced by ~61%, ~64%, ~62% and ~82%, respectively. (B) Same as before except, the ImageJ analysis represents Fig1F. Data represent mean of at least three biological experiments (± SD).

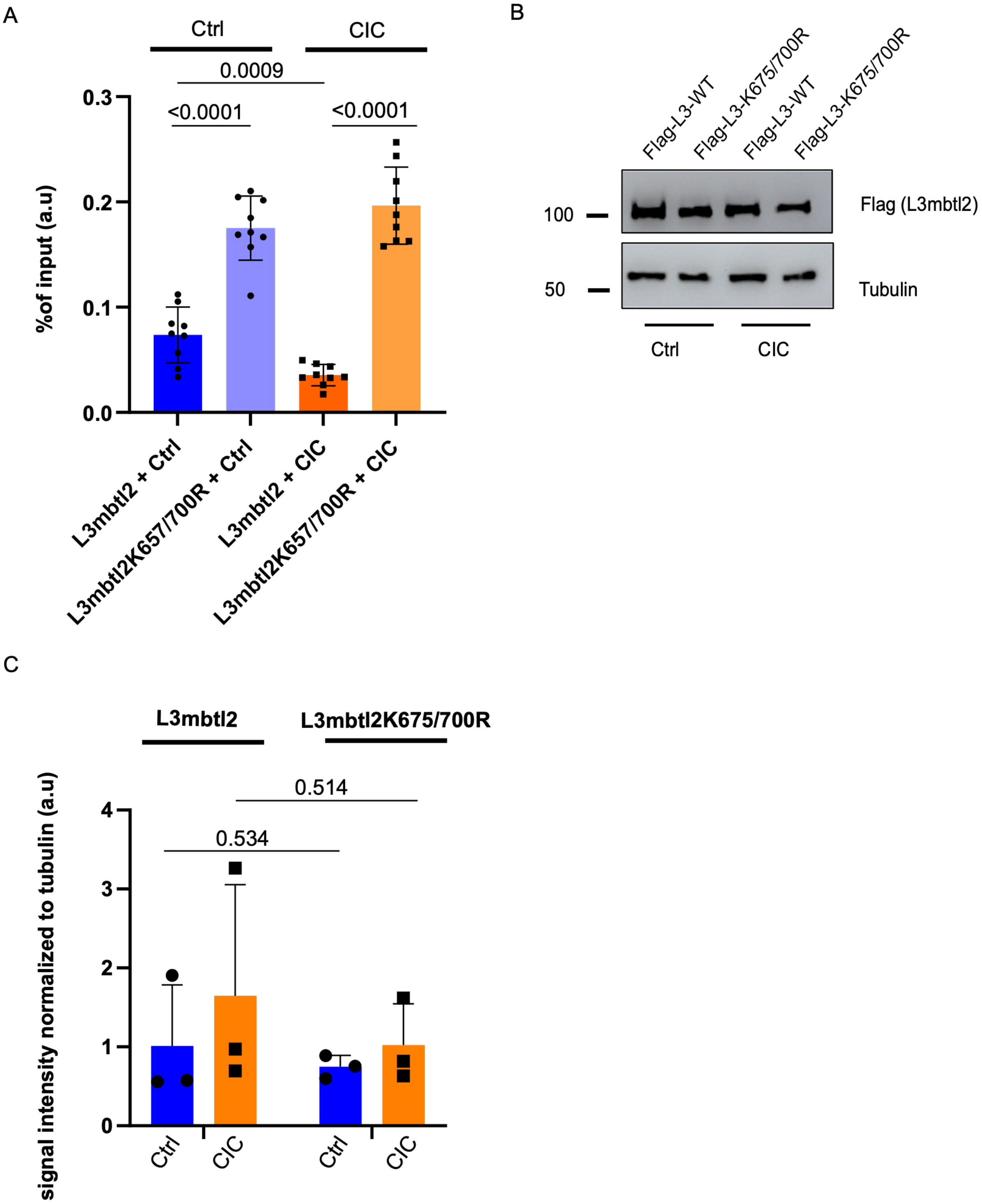

Figure S10 (related to Figure 4): (A) Similar to Figure 4A except, chromatin recruitment Flag-L3-WT and Flag-L3-K675/700R is shown. 5 µg of plasmid was transfected on day 2 of differentiation muscle cells using TransIT reagent (Mirus) according to the manufacturer's protocol. CIC was induced on day 3 for 48 hrs. Data represents mean value of three independent experiments with 9 technical replicates. (B) Representive western blot shows expression of Flag-L3 proteins. (C) ImageJ analysis shows relative amount of indicated Flag-L3 protein signals (nornalized against tubulin) from three independent experiments.

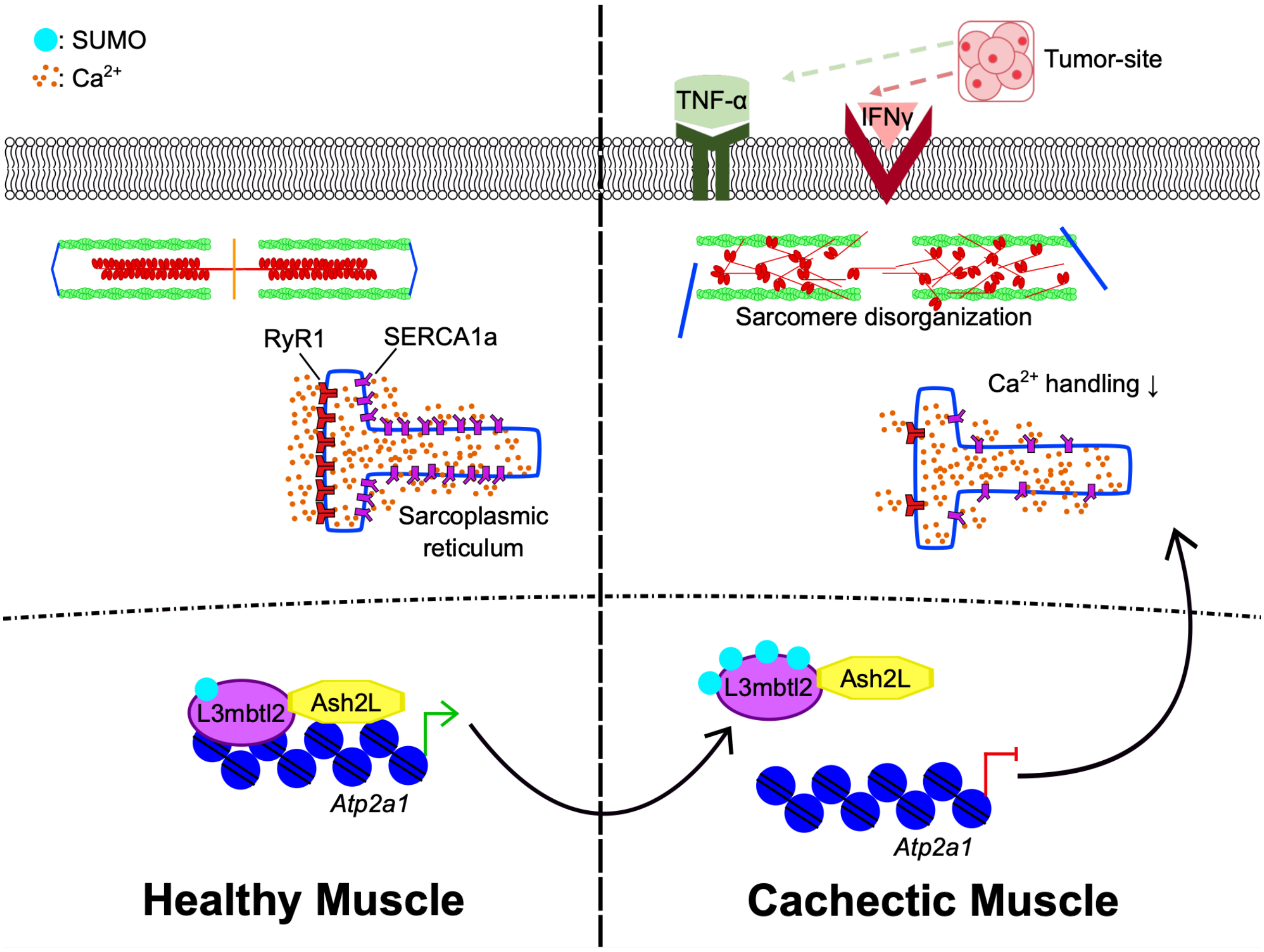

Figure S11: Schematic representation of our working model depicting molecular mechanisms of muscle contractile dysfunction underlying CIC. In control cell, L3mbtl2 and Ash2L present on the promoter of *Atp2a1* thereby ensure correct transcription signalling and proper expression of muscle-specific genes particular *Atp2a1*, critical for proper muscle function. Increased SUMOylation of L3mbtl2 in CIC leads to detachment of L3mbtl2 and Ash2l from *Atp2a1,* leading to muscle dysfunction.

Supplementary movie and table legends

Supplementary video S1 Contractile ability of control siRNA-treated (left) and L3mbtl2 siRNA-treated cells (right)

Myotubes on coverslips immersed in differentiation medium and electrically paced at 40 V, 1 Hz, and 4 ms pulse duration was recorded at 15 frames/sec for 15 seconds. Movie is played at 15 frames/sec. Scale bar corresponds to distance of 50 µm.

Supplementary table1: (related to Figure 2) Processed data from mass spectrometric proteomics analysis after endogenous SUMO2/3-immunoprecipitation. Data after analysis with MaxQuant and Perseus. PGs and their changed SUMOylation in CIC, as well as changes for each replicate are shown. Sheet1 – Proteins with upregulated SUMOylation. Sheet 2 - Proteins with downregulated SUMOylation.
