# Supplementary material for: Reorientation of the SUMOylation landscape and altered L3mbtl2-mediated transcriptional activity in cancer-associated muscle contractile dysfunction
Source: Cell Death Discov. 2026 Sep 9;12:360. doi: 10.1038/s41420-026-03337-y (PMC13558696; doi:10.1038/s41420-026-03337-y)
Supplement: Supplementary file 2 [file 41420_2026_3337_MOESM4_ESM.pdf]

Original blots (SUMO2/3) of **Figure 1A**

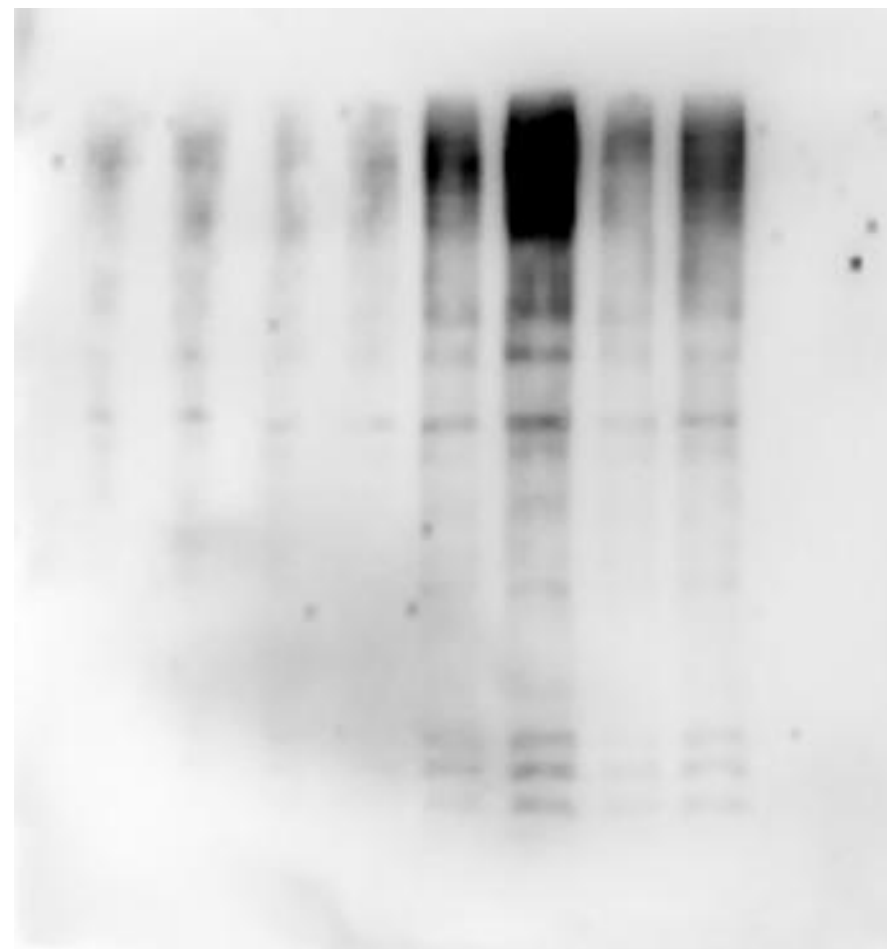

Original blot (RNA Pol II) of **Figure 1A**

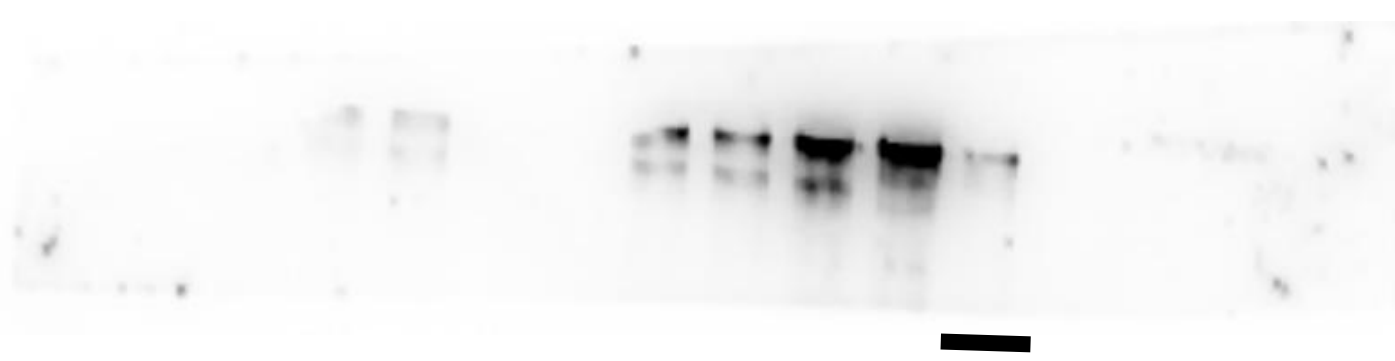

This lane is **not** part of the figure

Original blots (Ash2L) of **Figure 1A**

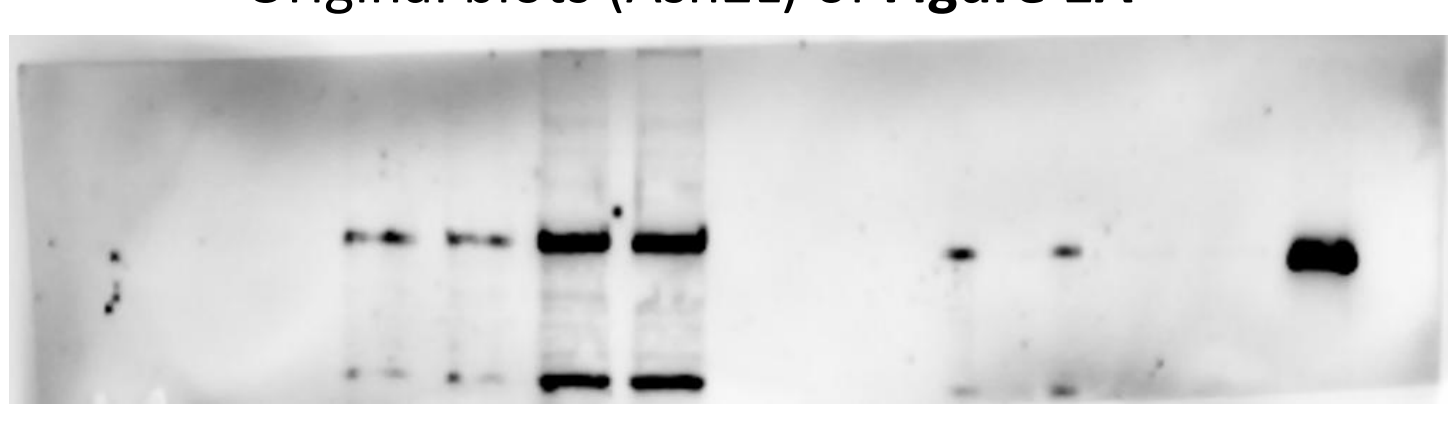

These lanes are the part of the figure.
Note, loading was in reverse direction

Original blots (Tublulin) of **Figure 1A**

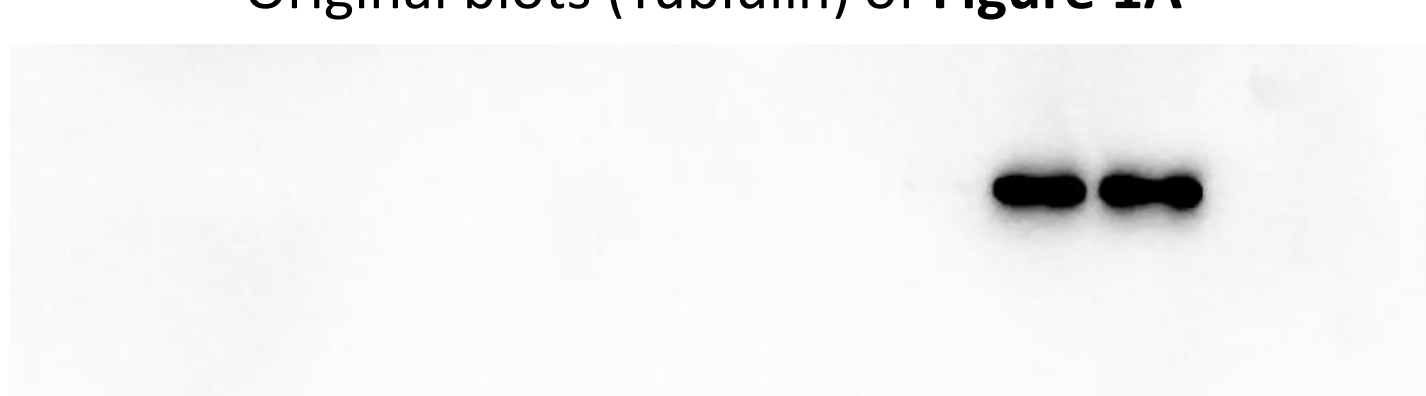

. **Note, loading was in reverse direction**

Original blots (Tubulin) of **Figure 1E**

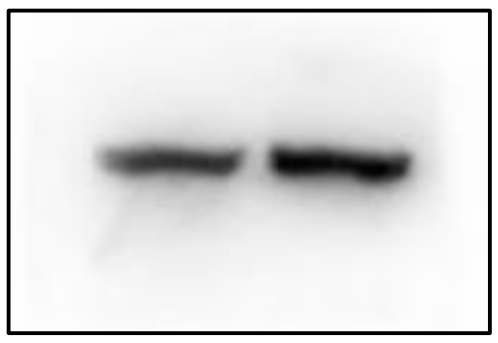

Original blots (Senp1) of **Figure 1E**

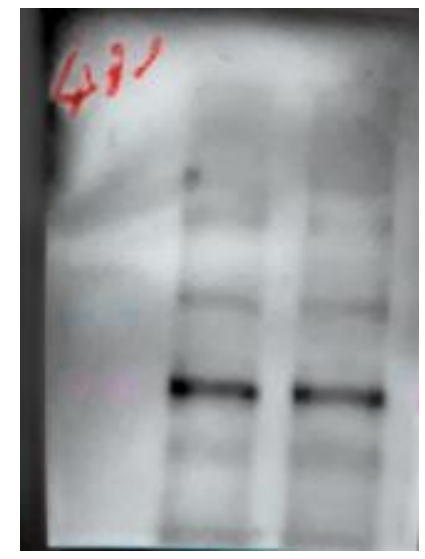

Original blots (Senp3) of **Figure 1E**

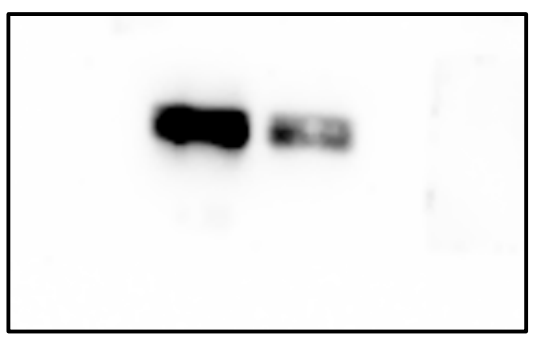

Original blots (Senp5) of **Figure1E**

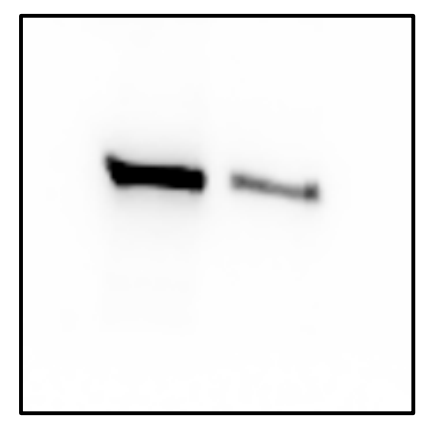

Original blots (Senp6) of **Figure 1E**

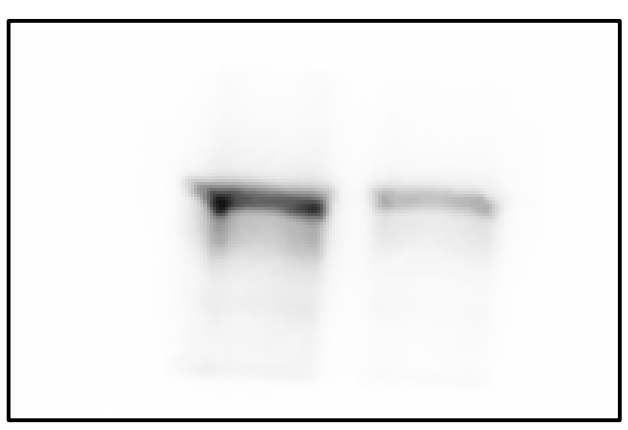

Original blots (Senp7) of **Figure 1E**

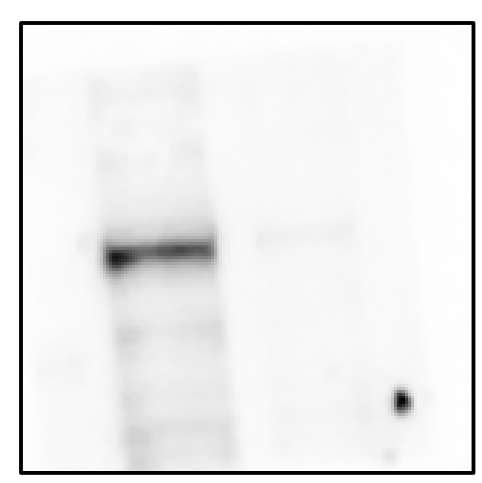

Original blots (Senp1) of **Figure 1F**

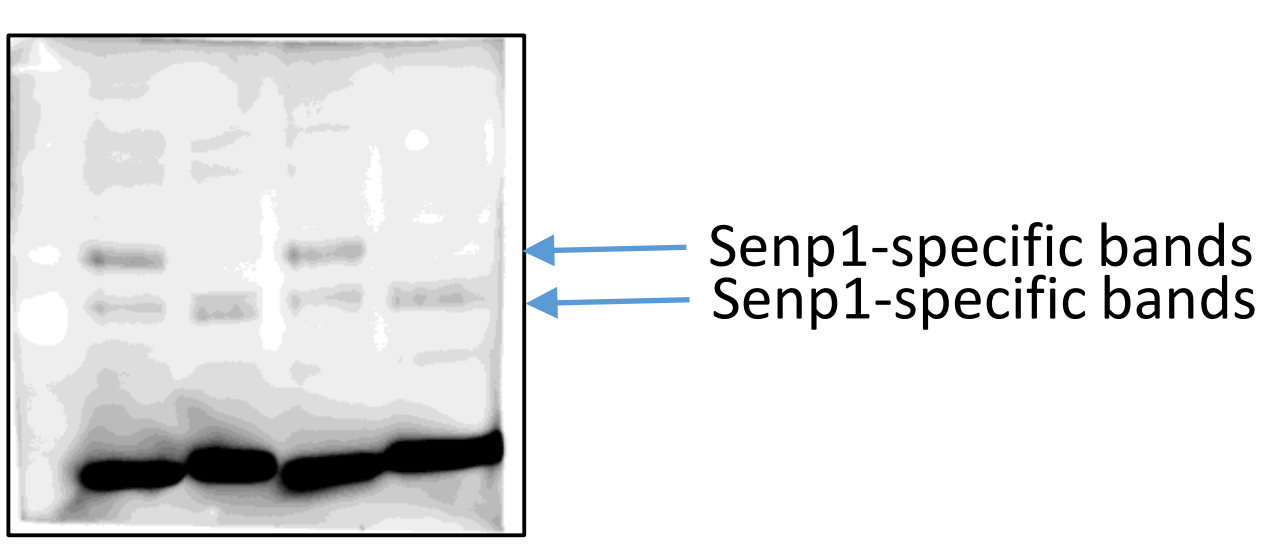

Original blots (Tubulin) of **Figure 1F**

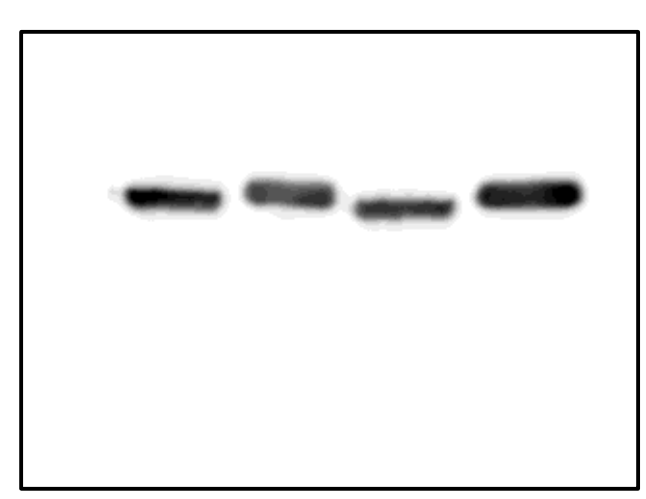

Original blots (L3mbtl2) of **Figure 3G**

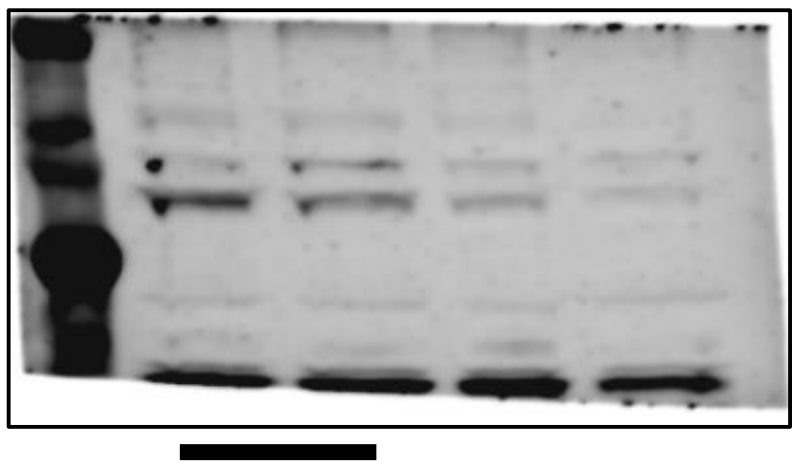

These two lanes are the part of the figure

Original blots (Tubulin_1) of **Figure 3G**

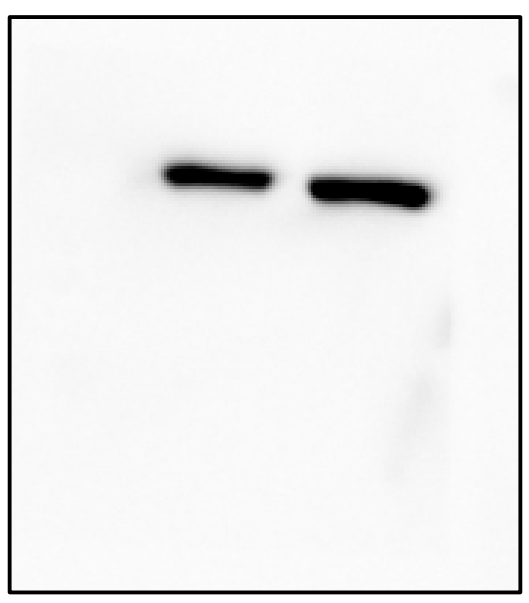

Original blots (MyHC-IId) of **Figure 3G**

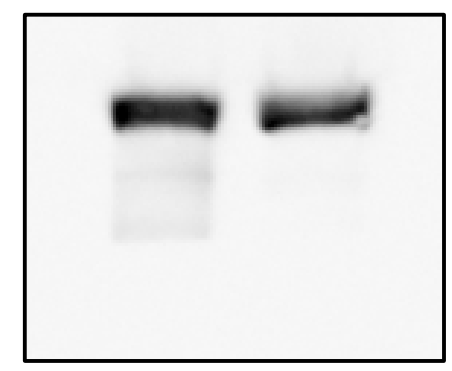

Original blots (SERCA1) of **Figure 3G**

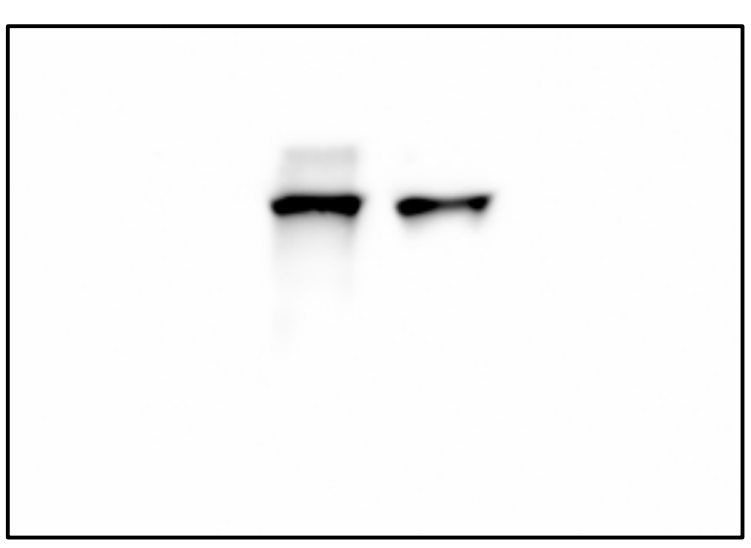

Original blots (Tubulin_2) **of Figure 3G**

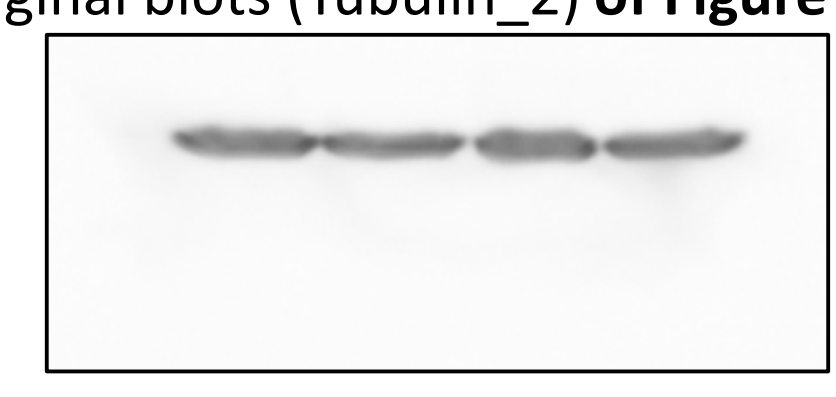

These two lanes are the part of the figure

Original blots (MHC-Iid_Input) of **Figure 4B**

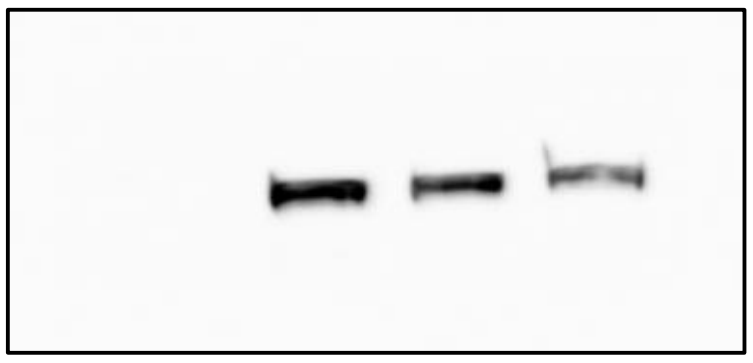

Original blots (Ash2L_Input) of **Figure 4B**

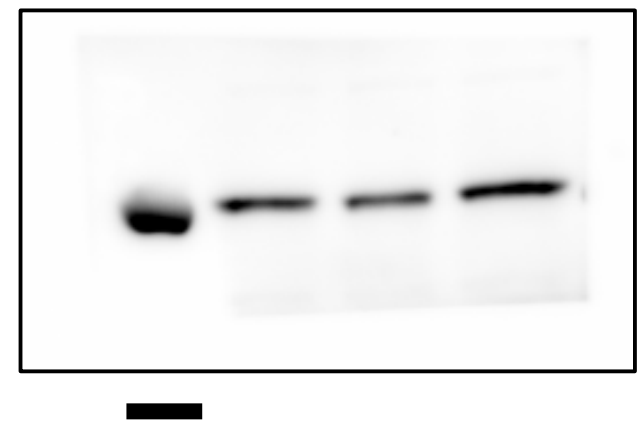

This lane is **not** part of the figure

Original blots (Pcgf6_Input) of **Figure 4B**

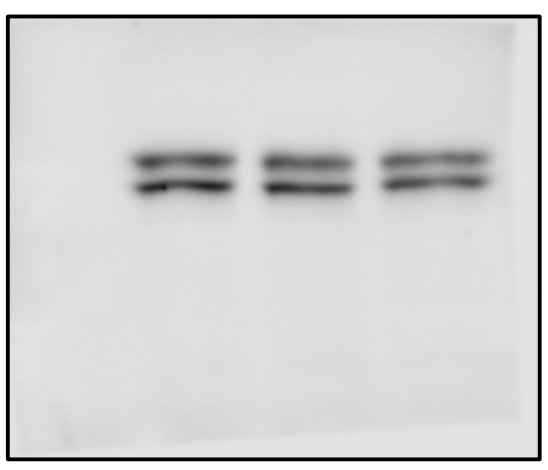

Original blots (Flag-L3mbtl2_Input) of **Figure 4B**

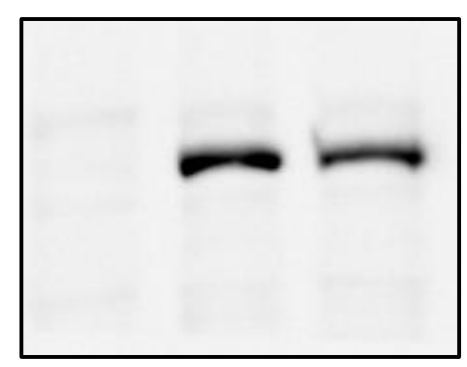

Original blots (Tubulin) of **Figure 4B**

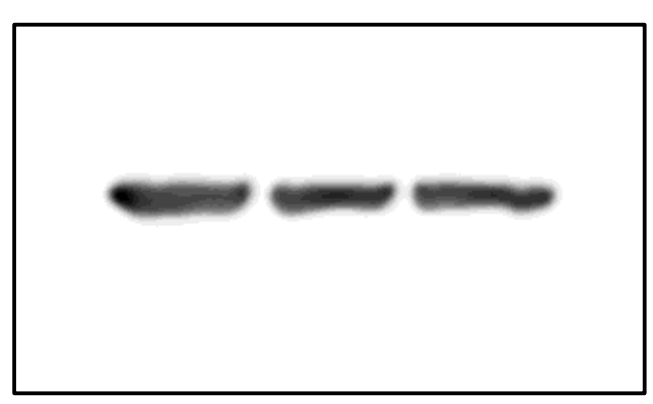

Original blots (Flag-L3mbtl2_from IP) of **Figure 4B**

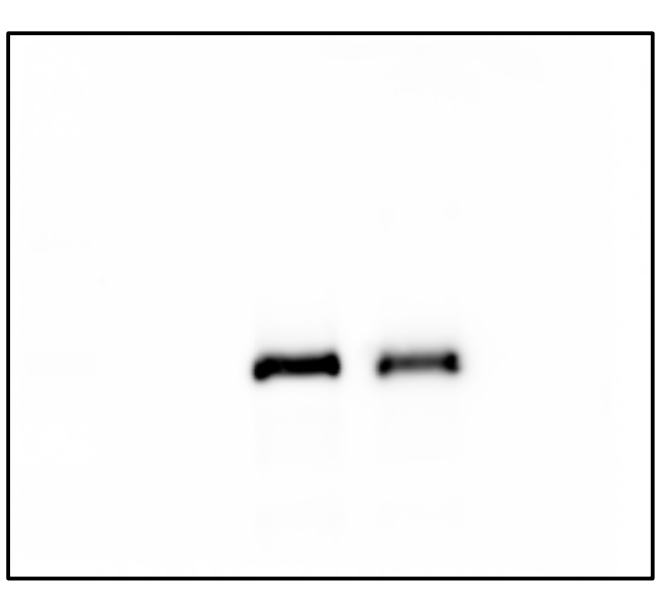

Original blots (Pcgf6_from IP) of **Figure 4B**

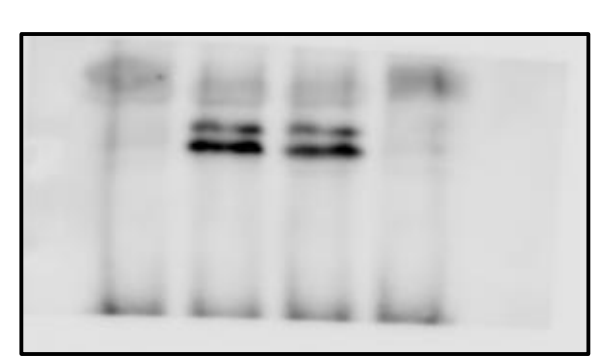

This lane is **not** part of the figure

Original blots (Ash2l_from IP) of **Figure 4B**

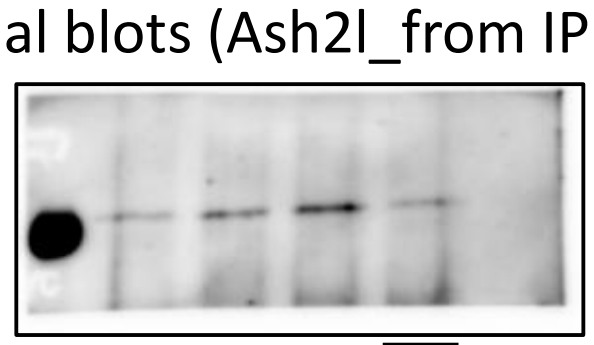

This lane is **not** part of the figure

Original blots (SUMO2/3) of **Figure S1B**

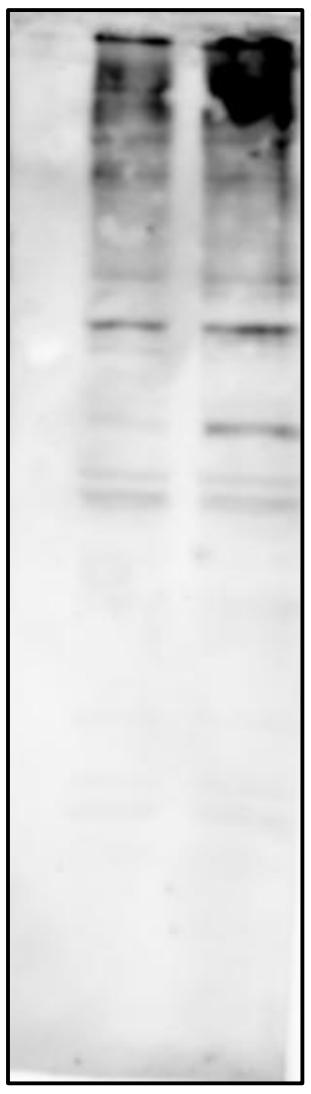

Original blots (Tubulin_1) of **Figure S1B**

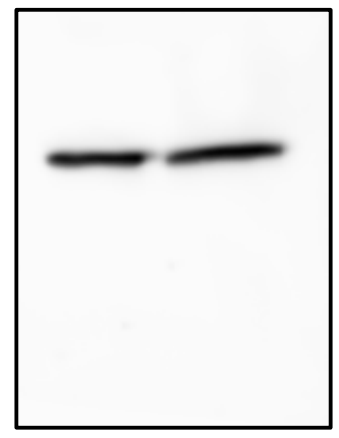

Original blots (Myhc-iid) of **Figure S1B**

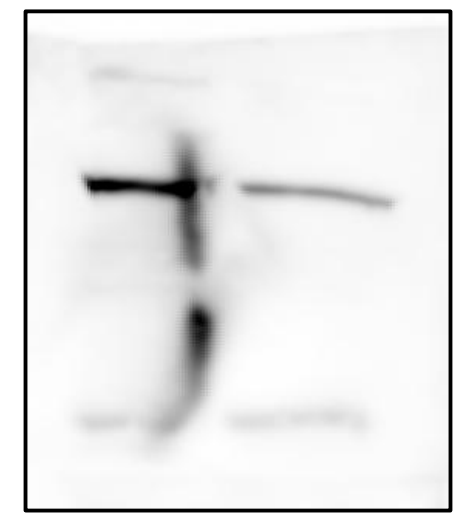

Original blots (SUMO1) of **Figure S1B**

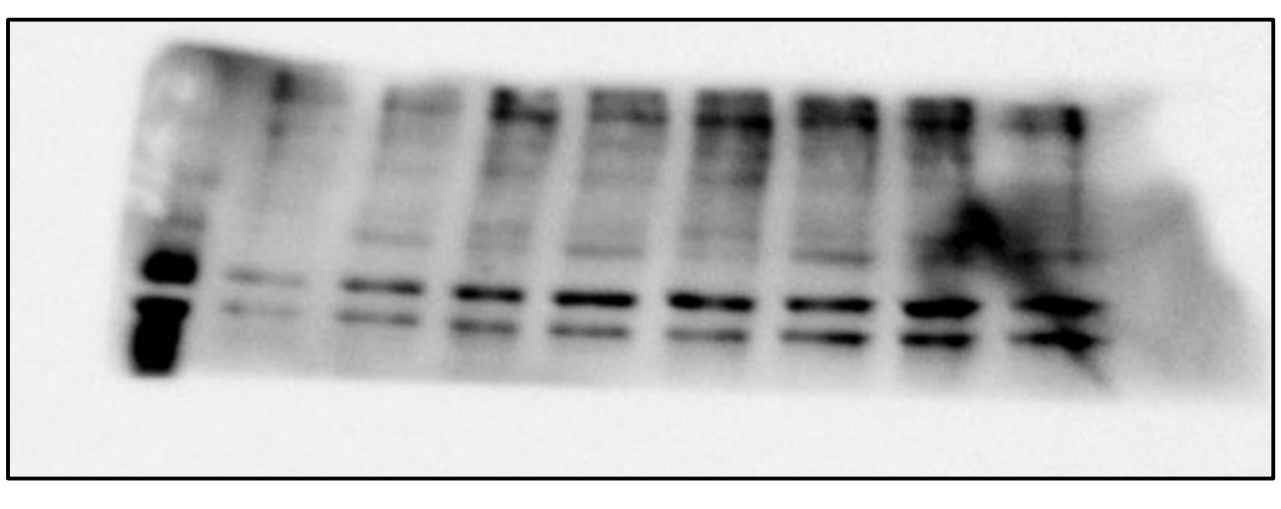

These two lanes are the part of the figure

Original blots (Tubulin_2) of **Figure S1B**

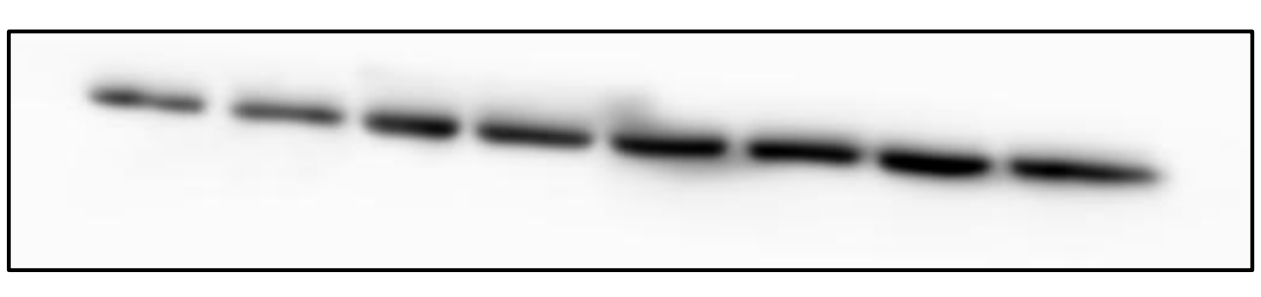

These two lanes are the part of the figure

Original blots (SUMO2/3) of **Figure S1C**

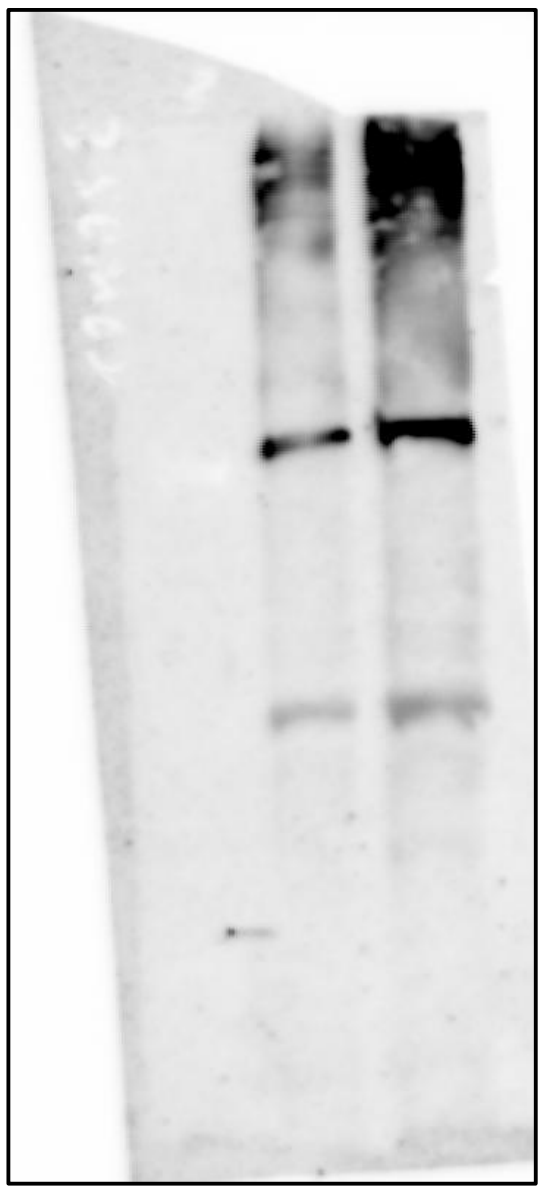

Original blots (Tubulin) of **Figure S1C**

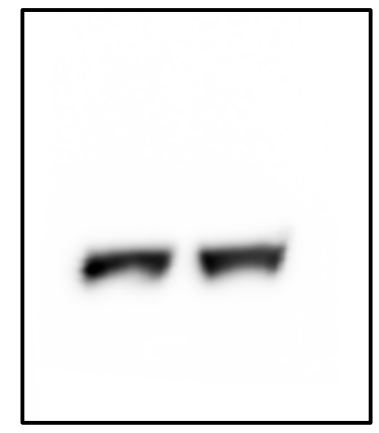

Original blots (MyHC-IId) of **Figure S1C**

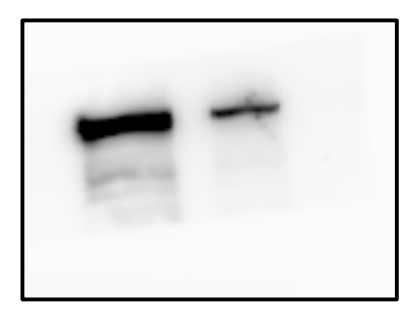

Original blots (SUMO1) of **Figure S1C**

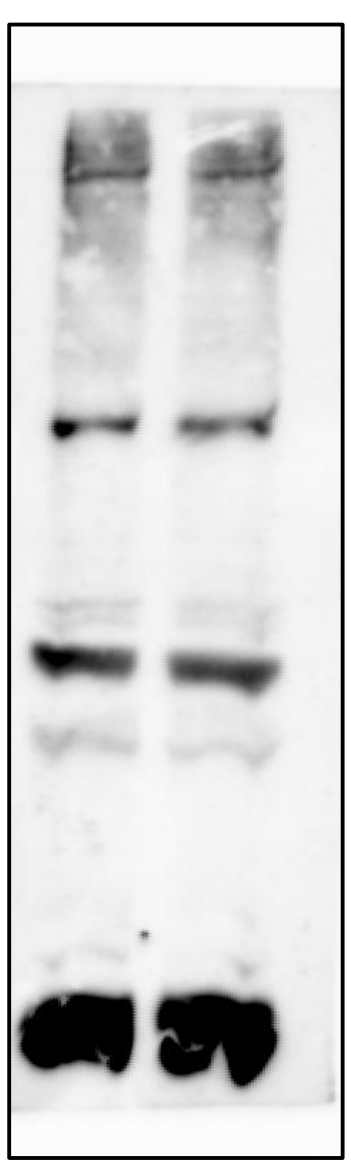

Original blots (SUMO2/3) of **Figure S1D**

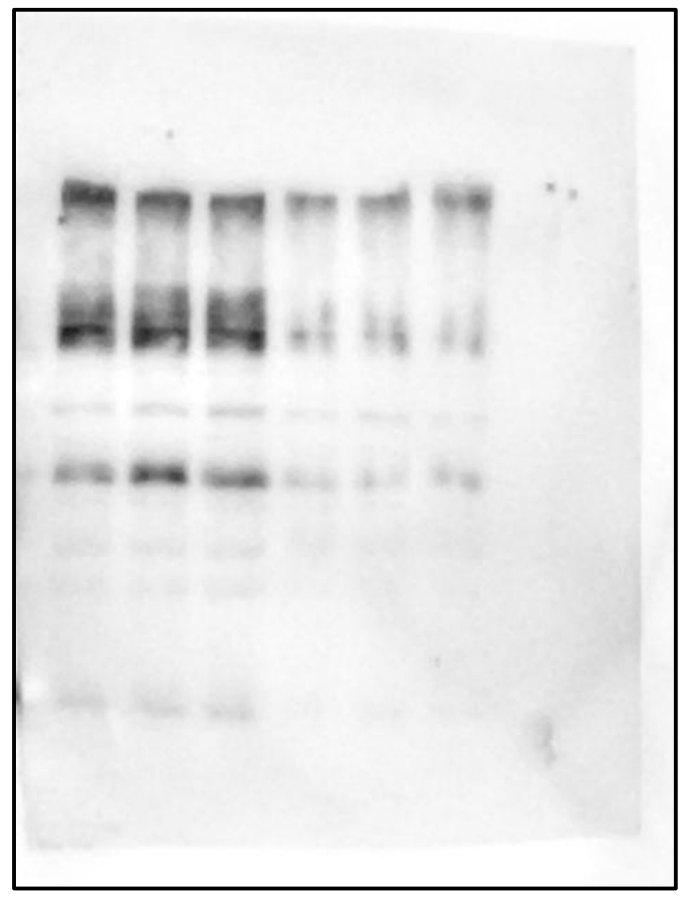

Please note the loading sequence – three lanes from the left represent lysates orginated from cachectic mice. Three lanes from right represent lysates orginated from control mice

Original blots (MyHC-IId) of **Figure S1D**

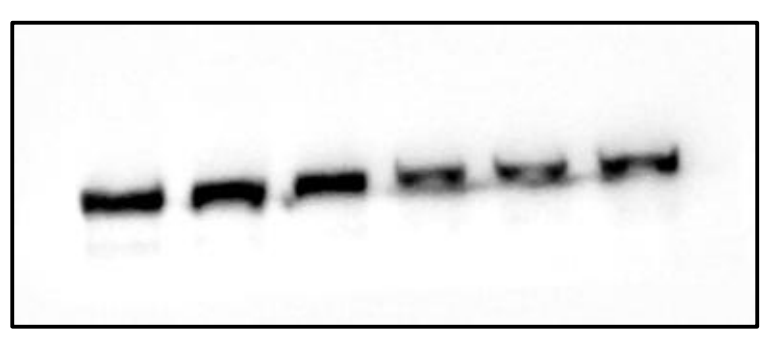

Please note the loading sequence – three lanes from left represent lysates orginated from control mice. Three lanes from right represent lysates orginated from cachectic mice.

Original blots (Tubulin) of **Figure S1D**

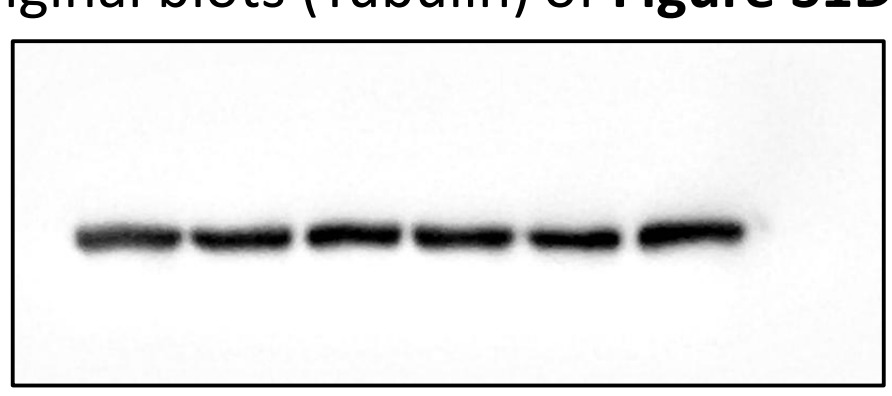

Please note the loading sequence – three lanes from left represent lysates orginated from control mice. Three lanes from right represent lysates orginated from cachectic mice.

Original blots (Myhc-iid) of **Figure S2A**

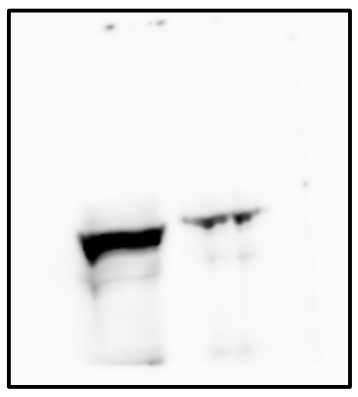

Original blots (L3mbtl2) of **Figure S2B**

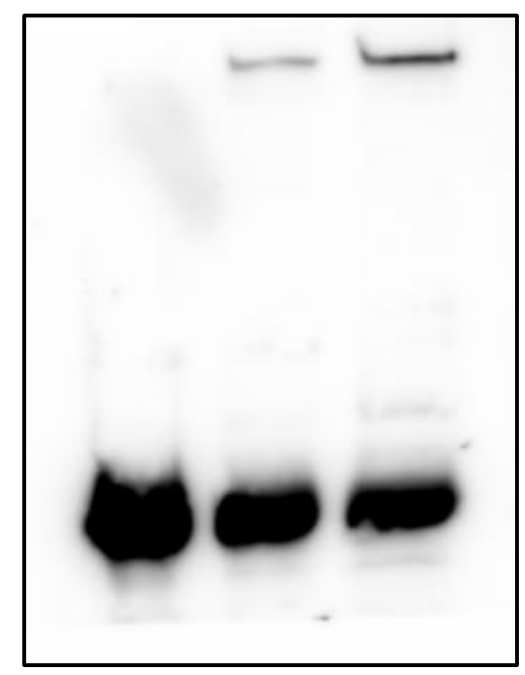

Original blots (L3mbtl2) of **Figure S2A**

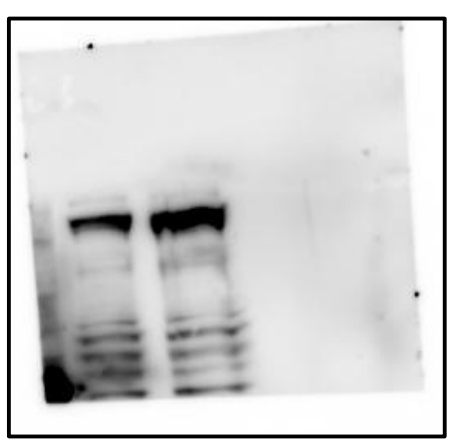

Original blots (Tubulin) of **Figure S2A**

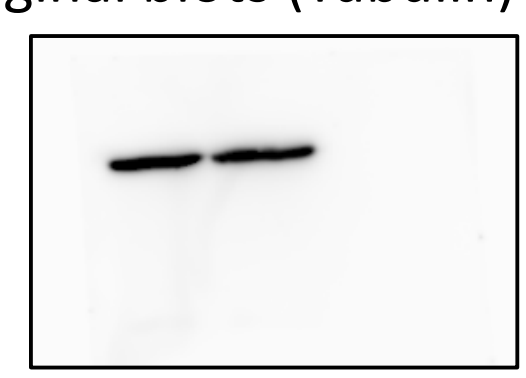

Original blots (Flag) of **Figure S10B**

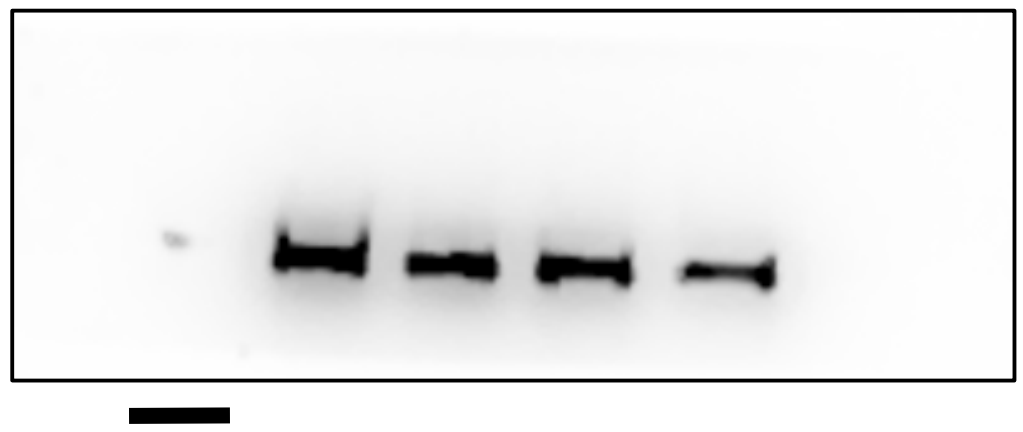

This lane is not the part of the figure.

Original blots (Tubulin) of **Figure S10B**

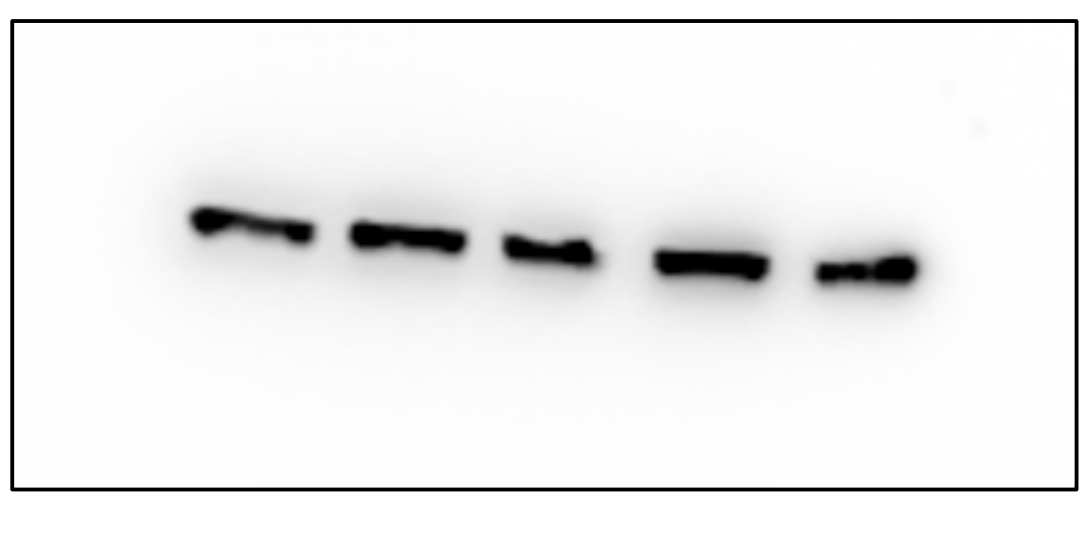

This lane is not the part of the figure.
